# Epithelial SLPI expression in severe inflammatory bowel disease relates to high IL-17 and neutrophil programming

**DOI:** 10.1172/jci.insight.197126

**Published:** 2026-03-10

**Authors:** Sandrine Nugteren, Beatriz Calado, Ytje Simons-Oosterhuis, Daniëlle H. Hulleman-van Haaften, Willem K. Smits, Renz C.W. Klomberg, Bastiaan Tuk, Mohammed Charrout, Dicky J. Lindenbergh-Kortleve, Michail Doukas, Mathijs A. Sanders, Gregory van Beek, Johanna C. Escher, Lissy de Ridder, Maria Fernanda Pascutti, Janneke N. Samsom

**Affiliations:** 1 Laboratory of Pediatrics, Division Gastroenterology and Nutrition, Erasmus University Medical Center, Rotterdam, Netherlands.; 2 Department of Pediatric Gastroenterology, Sophia Children’s Hospital–Erasmus University Medical Center, Rotterdam, Netherlands.; 3 Delft Bioinformatics Lab, Delft University of Technology, Delft, Netherlands.; 4 Department of Pathology, Erasmus University Medical Center, Rotterdam, Netherlands.; 5 Department of Hematology, Erasmus MC Cancer Institute, Erasmus University Medical Center, Rotterdam, Netherlands.

**Keywords:** Gastroenterology, Immunology, Inflammatory bowel disease

## Abstract

Heterogeneity in disease severity and treatment response in inflammatory bowel disease (IBD) likely evolves from individual differences in host-microbiota-immune interactions. Histological evaluation of intestinal biopsies is central to diagnosis, but histological parameters that define underlying immune mechanisms are limited. We investigated histological features that distinguish individual patient immune profiles in therapy-naive pediatric IBD patients (age 6–18 years) using biopsy immunohistochemistry and transcriptomics and plasma proteomics across two cohorts. High colonic epithelial expression of secretory leukocyte protease inhibitor (SLPI), a microbiota-induced regulator of epithelial function, occurred in IBD patients with high clinical disease activity and more severe endoscopic and microscopic disease activity. SLPI expression was related to increased neutrophil infiltration, transcriptomic signatures of activation, and genes known to associate with therapeutic resistance. High SLPI colocalized with high densities of IL-17–secreting cells and was associated with high plasma concentrations of Th17-related immune proteins. Additionally, patients with high intestinal SLPI had an intrinsically different immunotype, in which circulating neutrophils exhibited altered transcription of genes involved in neutrophil granule formation, phagocytosis, oxidative phosphorylation, and interferon signaling. Thus, high colonic SLPI expression at diagnosis associates with severe IBD, increased IL-17A–neutrophil pathway responses, and altered transcriptomic wiring of circulating neutrophils.

## Abstract 2

Elevated expression of one single parameter, colonic SLPI, at diagnosis identifies Inflammatory Bowel Disease patients with severe disease and immune features of therapeutic resistance.

## Introduction

Chronic gastrointestinal inflammation in inflammatory bowel disease (IBD) is driven by aberrant host immune responses to harmless microbial antigens. Cases are classified based on clinical and pathological characteristics into one of the two major clinical forms of IBD, Crohn’s disease (CD) and ulcerative colitis (UC), which have different treatment strategies.

However, within these clinical subtypes, the disease is heterogeneous with highly varied responses to therapy and disease courses. If insufficient control of inflammation is achieved, patients can develop complications such as strictures and may require surgery. Anti–tumor necrosis factor-α (anti–TNF-α) biologicals can induce remission in severe relapsing pediatric IBD, but about 40% of cases do not respond to or become refractory to anti–TNF-α in the long term (1–4). Although IBD is an immune-mediated disease, robust immune parameters that enable precise classification of the heterogeneity of the underlying immune disease, predict the clinical disease course, or allow development of tailored treatments for the underlying immune dysfunction are lacking (5). New developments in systems analysis allow comprehensive interrogation of complex immune responses, but require simplification for applicability in clinical practice (6, 7). Histological evaluation of intestinal biopsies is a key step in the diagnosis of IBD. Therefore, identifying histological parameters that differentiate the heterogeneous underlying intestinal immune processes at diagnosis could potentially enable more precise immune classification of IBD.

Recent evidence indicates that neutrophils contribute to a complicated disease course and therapeutic resistance in IBD. Persistent histological disease activity, specifically tissue neutrophil infiltration despite endoscopically normal mucosa, predicts relapse in UC (8, 9). Interestingly, high expression of oncostatin M (*OSM*), a neutrophil product, in the inflamed intestine is associated with severe histological disease and non-response to anti–TNF-α in IBD (10, 11). However, neutrophils are a heterogeneous population that exert highly varied immune activities, and the factors that explain why neutrophil recruitment and activation are associated with poor disease outcome in IBD are currently unclear (12). Microbial-epithelial interactions and subsequent production of cytokines and chemokines by epithelial cells and fibroblasts orchestrate neutrophil infiltration and function. Therefore, investigation of epithelial function in intestinal biopsies at diagnosis could help to clarify the epithelial-neutrophil-inflammation axis in individual IBD patients.

We previously discovered that secretory leukocyte protease inhibitor (SLPI) regulates the production of chemokines in the intestinal epithelium in response to microbiota (13). SLPI is mainly expressed by epithelial cells in the gastrointestinal and respiratory tract (14, 15) and prevents tissue damage by inhibiting proteases, including neutrophil elastase (16). SLPI also inhibits nuclear factor-κB–mediated (NF-κB–mediated) inflammatory gene transcription and thereby regulates the threshold of innate immune responses to microbial signals (17–19). Moreover, SLPI exerts antibacterial, antifungal, and antiviral properties (20–23). In the intestinal epithelium, SLPI expression is more prominent in the colon than in the small intestine and is induced by repetitive microbial contact, as observed during colonization of germ-free mice (13). Therefore, we hypothesized that high intestinal epithelial SLPI expression reflects increased contact between epithelial cells and microorganisms. As SLPI neutralizes neutrophil elastase and suppresses production of neutrophil attractant C-X-C motif chemokine ligand 8 (CXCL8) (13), we anticipated that epithelial SLPI expression would be upregulated in tissues heavily infiltrated by inflammatory neutrophils. Therefore, we examined whether epithelial SLPI expression varies in intestinal biopsies from patients with pediatric IBD and assessed whether high SLPI expression at diagnosis associates with a distinctive immune response and distinct subtype of clinical disease.

Here, we demonstrate that immunohistochemical detection of epithelial SLPI protein in colonic biopsies associates with a distinctive immune profile in pediatric IBD at diagnosis, irrespective of whether patients have CD or UC. Patients with high intestinal SLPI protein expression exhibited a higher endoscopic severity of inflammation and increased histological and clinical disease activity. Histology revealed that high SLPI expression colocalized with abundant neutrophil infiltration. Paired-biopsy mRNA analyses demonstrated a signature of neutrophilic inflammation in SLPI^high^ biopsies, including upregulation of *OSM*, *IL1B*, and *CXCL8*, which have previously been associated with severe disease progression and resistance to anti–TNF-α (10, 11). Crucially, circulating neutrophils from SLPI^high^ patients were intrinsically different, exhibiting altered transcription of genes involved in neutrophil granule formation, phagocytosis, oxidative phosphorylation, and interferon signaling. Furthermore, this high SLPI-inflammatory neutrophil network may be amplified by T cell–derived interleukin-17A (IL-17A), as we found that IL-17A stimulated SLPI expression in epithelial cells in vitro and was more abundant in the intestinal lesions and circulation of SLPI^high^ patients.

## Results

### SLPI expression is upregulated in the colonic epithelium in two experimental murine models of colitis.

**Figure 1:**
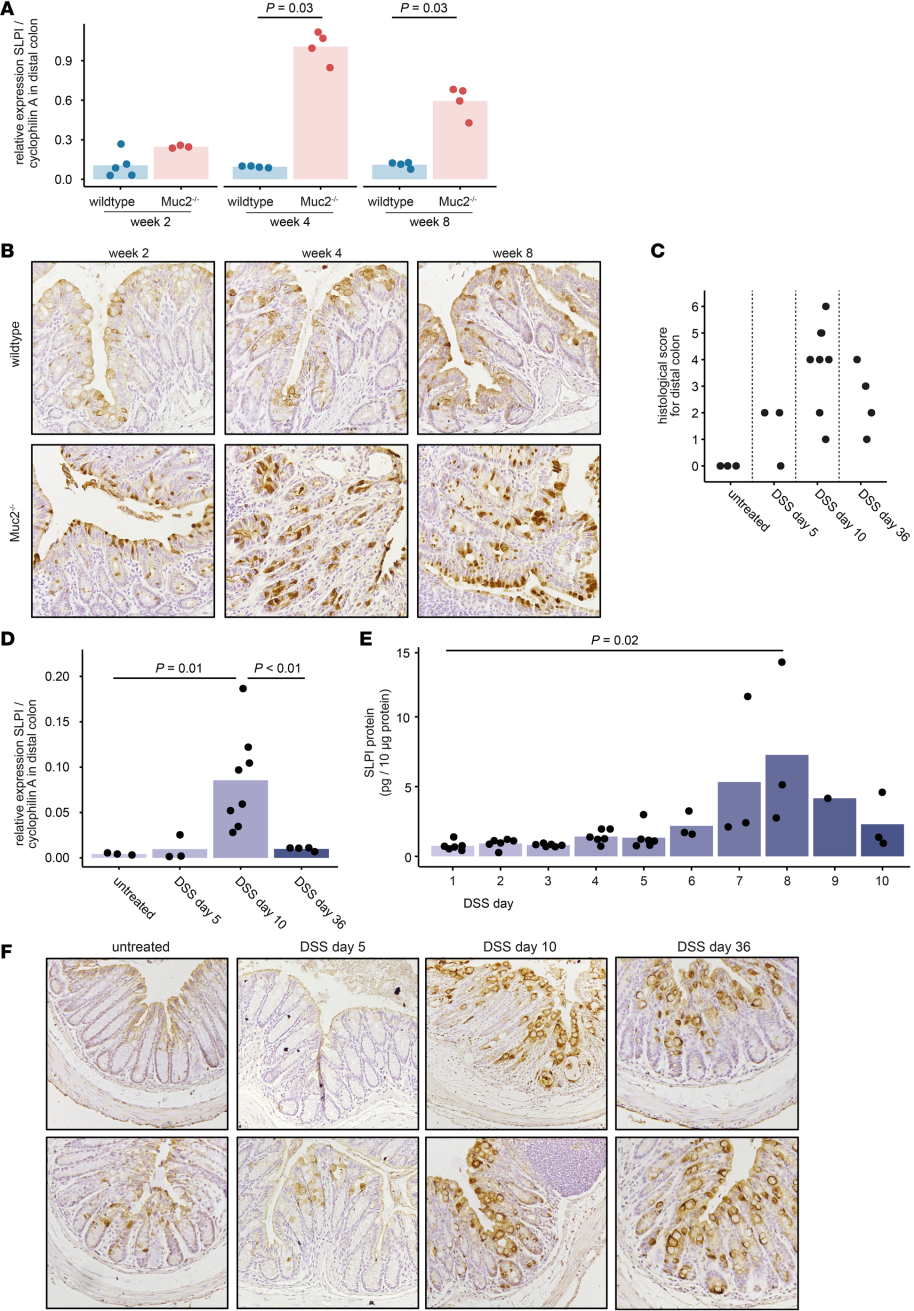
SLPI expression is upregulated in the distal colon of Muc2^–/–^ mice, and in the distal colon of wild-type mice during DSS-induced colitis. (**A**) *Slpi* mRNA expression was measured in the distal colonic tissue of *Muc2*-deficient (Muc2^–/–^) mice and wild-type littermates at 2, 4, and 8 weeks old. Bars represent the mean relative expression levels for multiple mice; *P* values were calculated using Wilcoxon’s rank sum test (**A**, **D**, and **E**). (**B**) SLPI protein expression was detected by immunohistochemistry (IHC) in the distal colonic tissue of Muc2^–/–^ mice and wild-type littermates at 2, 4, and 8 weeks old. Representative images were acquired at ×20 magnification. (**C**–**F**) Wild-type mice received 2% DSS in the drinking water for 5 subsequent days in 2 independent experiments. (**C**) Histological damage and inflammation were scored on a scale from 0 to 6 on H&E-stained sections of the distal colon. (**D**) *Slpi* mRNA expression was measured in the distal colon of untreated mice and at day 5, day 10, and day 36 of DSS treatment. (**E**) SLPI protein excretion was measured in fecal samples collected on day 1 until day 10 of DSS treatment. Shown are SLPI protein levels relative to total protein levels for mice from which fecal samples were available on that day. Bars represent the mean relative SLPI protein levels for multiple mice. (**F**) SLPI protein expression was detected by IHC in the distal colon of untreated mice and at day 5, day 10, and day 36 of DSS treatment. Representative H&E and SLPI-immunostained images of the distal colon of 2 mice per condition; original magnification, ×10.

Epithelial SLPI expression is regulated by microbial interactions and is more prominent in colon than small intestine (13, 24, 25). Therefore, we questioned whether increased contact between epithelial cells and microorganisms, as occurs in IBD, upregulates epithelial SLPI expression. First, we assessed colonic SLPI expression in Muc2-deficient (Muc2^–/–^) mice. Muc2, the main secretory mucin expressed by the human and murine colonic epithelium, is an important component of the intestinal mucus layer (26, 27). In Muc2^–/–^ mice, bacteria are in close contact with the epithelium and enter deep into crypts (28), which results in spontaneous colitis (29). We observed significantly higher *Slpi* mRNA expression in the distal colon of 4- and 8-week-old Muc2^–/–^ mice compared with wild-type littermates (Figure 1A). In addition, epithelial cells in the distal colon of 2-, 4-, and 8-week-old Muc2^–/–^ mice exhibited more intense SLPI protein expression in comparison with wild-type (Figure 1B). SLPI was most abundant in large cells scattered throughout the epithelium (Figure 1B), in agreement with reports demonstrating that SLPI is produced by intestinal goblet-type epithelial cells (15).

To further investigate the relationship between SLPI, microbiota, and colitis, we assessed epithelial SLPI expression during dextran sodium sulfate–induced (DSS-induced) colitis, which occurs as a result of compromised barrier integrity leading to exposure to luminal antigens (30). Induction of DSS colitis in wild-type mice resulted in tissue damage and inflammation in the distal colon, with high histological disease scores on day 10 of DSS treatment (Figure 1C). DSS significantly increased *Slpi* mRNA expression in the distal colon of wild-type mice at day 10 (vs. untreated mice); *Slpi* expression returned to baseline levels at day 36 (Figure 1D). In addition, we detected a transient increase in SLPI protein concentrations in fecal samples (Figure 1E). Microscopically, we observed numerous SLPI-positive epithelial cells in the distal colon at days 10 and 36 in DSS-treated wild-type mice, whereas we only detected a few SLPI-positive cells in the distal colon at day 5 and in untreated mice (Figure 1F). The finding that SLPI expression remains high in tissues at day 36, when tissue healing has taken place, is intriguing as SLPI has been reported to have wound healing properties (31). SLPI-positive epithelial cells were mainly large goblet-type cells. Thus, SLPI expression increased in both the colonic tissue and feces at the peak of colitis, possibly in response to increased microbial contact.

### SLPI expression is increased in the colonic epithelium of therapy-naive pediatric patients with IBD.

Next, we investigated whether intestinal epithelial SLPI expression is increased during human intestinal inflammation using microarray data for *SLPI* mRNA expression in intestinal biopsies from 2 adult IBD cohorts from the Gene Expression Omnibus (GEO) public genomic repository.

**Figure 2:**
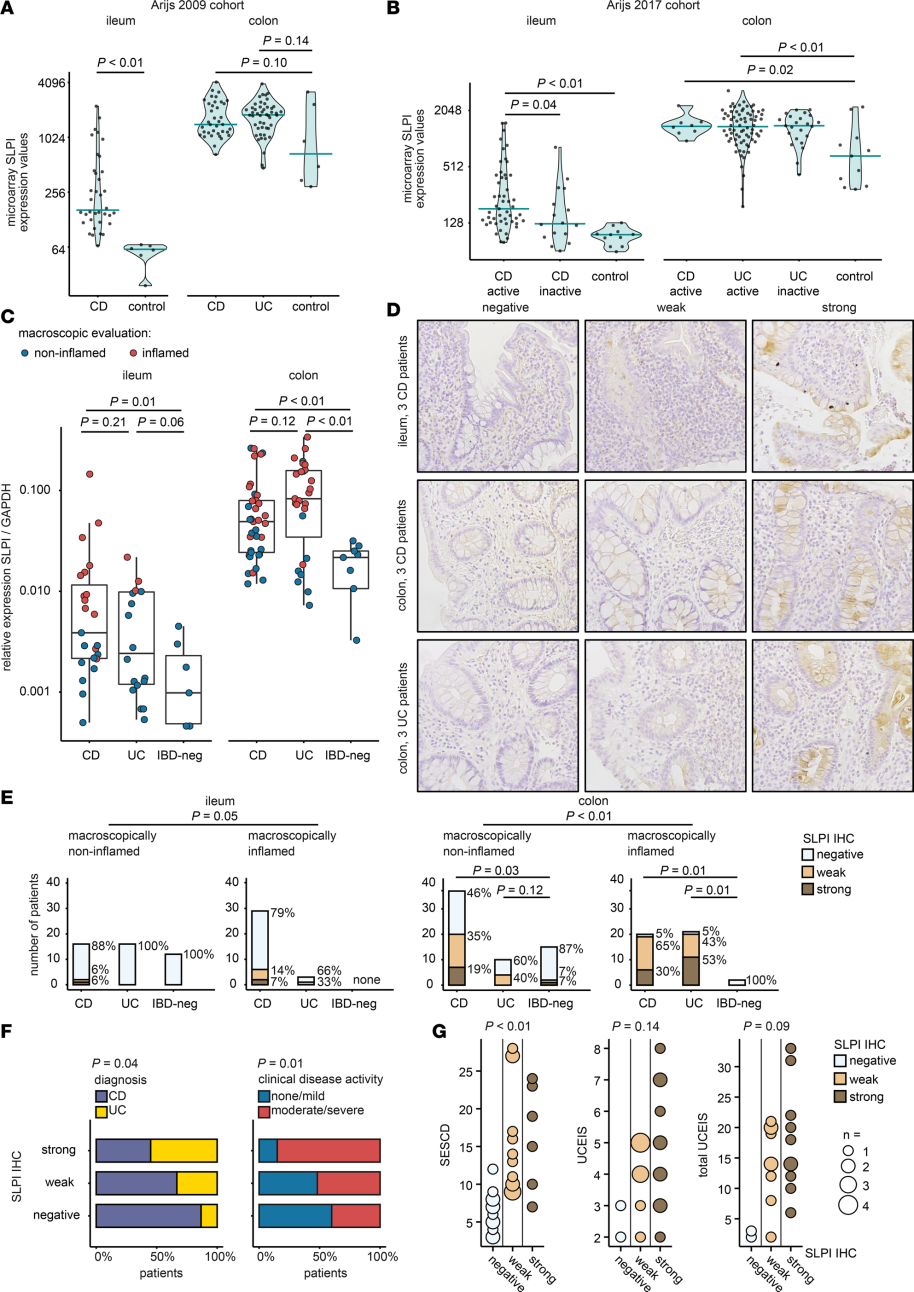
SLPI expression is increased in colonic epithelium of therapy-naive pediatric patients with CD or UC compared with IBD-negative controls. (**A** and **B**) *SLPI* RNA expression values from microarray data for intestinal biopsies from adult patients with CD or UC and controls. Data are derived from GSE16879 (Arijs 2009 cohort) (**A**) and GSE75214 (Arijs 2017 cohort) (**B**). Horizontal bars represent medians. Wilcoxon’s rank sum test. (**C**) *SLPI* mRNA expression measured by qPCR in macroscopically non-inflamed and macroscopically inflamed intestinal biopsies from pediatric CD (*n* = 21), UC (*n* = 19), and IBD-negative controls (IBD-neg, *n* = 9). Each data point represents one biopsy. Box plots display medians and first and third quartiles. Wilcoxon’s rank sum test. (**D** and **E**) SLPI protein expression detected by IHC in macroscopically non-inflamed and macroscopically inflamed intestinal biopsies from pediatric CD (*n* = 41), UC (*n* = 22), and IBD-negative controls (*n* = 15). (**D**) Representative images were acquired at ×20 magnification. (**E**) Intensity of intestinal epithelial SLPI staining scored semiquantitatively. Distribution of maximum SLPI scores and percentage of patients with each SLPI IHC score per group are shown. Fisher’s exact test. (**F** and **G**) Clinical characteristics per SLPI IHC score group using the SLPI IHC score for the colonic biopsy with the highest modified GHAS per patient. (**F**) Percentages of patients with CD (*n* = 40) versus UC (*n* = 22) and percentages of patients with no or mild clinical disease activity (*n* = 25) versus patients with moderate to severe clinical disease activity (*n* = 37). Pearson’s χ^2^ test. (**G**) SES-CD score in CD patients with a complete endoscopy (*n* = 31); UCEIS score in all UC patients (*n* = 22); and “total UCEIS” (the sum of the UCEIS of each colonic segment) in UC patients with a complete endoscopy (*n* = 21). The size of the circles represents the number of patients. Kruskal-Wallis rank sum test. For additional statistical comparisons, see Supplemental Table 2.

The Arijs 2009 cohort collected ileal and colonic biopsies from controls (healthy adults) and macroscopically inflamed mucosa from adult patients with IBD refractory to corticosteroids and/or immunosuppression (32). *SLPI* mRNA expression was significantly higher in colonic biopsies than ileal biopsies (Wilcoxon’s rank sum test, *P* < 0.01; Figure 2A). In addition, *SLPI* was significantly higher in ileal biopsies from patients with CD versus controls (Figure 2A). In colonic biopsies, *SLPI* tended to be higher in patients with CD or UC compared with controls, although these differences were non-significant (Figure 2A).

The Arijs 2017 cohort included ileal and colonic biopsies from controls (healthy adults) and endoscopically assessed adults with active (UC = Mayo endoscopic subscore ≥ 2; CD = presence of ulcers) or inactive IBD (33). *SLPI* was also higher in colonic biopsies than ileal biopsies in this cohort (Wilcoxon’s rank sum test, *P* < 0.01; Figure 2B). In addition, *SLPI* was higher in ileal biopsies from patients with active CD compared with controls and patients with inactive CD (Figure 2B). In colonic biopsies from patients with active CD or active UC, *SLPI* was also higher than controls (Figure 2B). *SLPI* was not different between colonic biopsies from patients with active and inactive UC as defined by endoscopy (Wilcoxon’s rank sum test, *P* = 0.73; Figure 2B). Collectively, these data indicate that *SLPI* mRNA expression is increased in the intestine of adult patients with IBD and may be associated with macroscopic disease activity.

The Arijs 2009 and Arijs 2017 cohorts were not therapy naive; thus, therapy could confound these results. Therefore, we assessed *SLPI* mRNA expression in ileal and colonic biopsies from therapy-naive pediatric patients with CD or UC and IBD-negative controls from our longitudinal pediatric IBD cohort. Overall, in both patients and IBD-negative controls, *SLPI* was higher in colonic biopsies than ileal biopsies (Wilcoxon’s rank sum test, *P* < 0.01). *SLPI* was also higher in biopsies from macroscopically inflamed regions than macroscopically non-inflamed regions (Wilcoxon’s rank sum test: ileum, *P* < 0.01; colon, *P* < 0.01; Figure 2C). *SLPI* was higher in both ileal and colonic biopsies from patients with CD (Figure 2C) and colonic biopsies from patients with UC versus IBD-negative controls (Figure 2C). *SLPI* was not significantly higher in ileal biopsies from patients with UC versus IBD-negative controls (Figure 2C), as most ileal UC biopsies were from macroscopically non-inflamed tissues. There was no significant difference in *SLPI* expression in inflamed colonic biopsies between CD and UC (Figure 2C). These data demonstrate that *SLPI* mRNA expression is increased in both the ileum and colon in untreated pediatric IBD and indicate that *SLPI* expression is associated with macroscopic inflammation.

To further explore whether histological detection of SLPI using immunohistochemistry could differentiate heterogeneous intestinal immune processes at diagnosis, we analyzed SLPI protein expression in the well-characterized Paediatric Inflammatory Bowel Diseases Network for Safety, Efficacy, Treatment and Quality improvement of care (PIBD-SETQuality) inception cohort, for which biological material is available for all patients included in Rotterdam (34, 35). First, we performed immunohistochemistry on ileal and colonic biopsies from therapy-naive pediatric IBD patients and IBD-negative controls using two different antibodies: a monoclonal antibody raised against human SLPI purified from sputum (Figure 2D) and a polyclonal antibody raised against *Escherichia coli*–derived recombinant human SLPI (Supplemental Figure 2A; supplemental material available online with this article; https://doi.org/10.1172/jci.insight.197126DS1). Detection of SLPI using the monoclonal antibody was significantly associated with detection of SLPI using the polyclonal antibody in both ileal and colonic biopsies from patients with IBD and IBD-negative controls (Fisher’s exact test: ileum, *n* = 76, *P* < 0.01; colon, *n* = 114, *P* < 0.01; Supplemental Figure 2B).

Regardless of the antibody used, we mainly detected SLPI in intestinal epithelial cells (Figure 2D and Supplemental Figure 2A). Consistent with our observations in mice, SLPI was most abundant in large goblet-like cells in the colon (Figure 2D and Supplemental Figure 2A). We compared SLPI protein expression between patients with IBD and IBD-negative controls using the maximum SLPI score for macroscopically inflamed and macroscopically non-inflamed ileum and colon (Figure 2E and Supplemental Figure 2C). SLPI protein expression was higher in colonic biopsies from macroscopically inflamed regions than in colonic biopsies from macroscopically non-inflamed regions (Fisher’s exact test, *P* < 0.01; Figure 2E and Supplemental Figure 2C). Importantly, SLPI was higher in colonic biopsies from macroscopically inflamed colon from patients with CD or UC compared with the IBD-negative controls (Fisher’s exact test, *P* = 0.01; Figure 2E and Supplemental Figure 2C). Moreover, therapy-naive patients with increased colonic SLPI expression had higher lipopolysaccharide-binding protein (LBP) concentrations in plasma, suggestive of increased barrier breach and intestinal permeability (Supplemental Figure 3). As the monoclonal antibody resulted in less background staining (Figure 2D and Supplemental Figure 2A), hereafter, we focus on the monoclonal antibody results.

To test whether colonic epithelial SLPI expression is related to clinical disease activity, we classified the patients based on the SLPI immunohistochemistry score for their most affected colonic biopsy, i.e., the highest modified Global Histological Disease Activity Score (GHAS).

Patients with UC had significantly higher SLPI scores than patients with CD (Pearson’s χ^2^ test, *P* = 0.04; Figure 2F and Supplemental Table 3). In 2 patients with UC, SLPI was not detected in the selected “most affected colonic biopsy” (Figure 2F); one of these patients had ulcerative proctitis without macroscopic inflammation in the colon. The second patient appeared to have mild disease; thus a macroscopically non-inflamed colon biopsy was selected based on the highest GHAS score (which was 3/12). Interestingly, significantly more IBD patients with high SLPI scores had moderate to severe clinical disease activity than no or mild clinical disease activity, indicating that high colonic epithelial SLPI protein expression is associated with more active clinical disease at diagnosis (Pearson’s χ^2^ test, *P* = 0.01; Figure 2F and Supplemental Table 3). Patients with CD with high SLPI scores more frequently had more severe endoscopic disease based on the Simple Endoscopic Score for Crohn’s Disease (SES-CD) (Figure 2G and Supplemental Table 3). As expected, patients with CD with high colonic SLPI scores more frequently had colonic or ileocolonic disease than ileal disease (Supplemental Table 3). In UC, we also observed trends between high SLPI scores and endoscopic disease based on the UC Endoscopic Index of Severity (UCEIS) or total UCEIS for each colonic segment, although these trends were non-significant (Figure 2G and Supplemental Table 3).

Next, we assessed whether high SLPI scores were related to the patient’s disease course. At weeks 2, 12, 24, and 52, approximately 50% of the patients with high colonic SLPI expression had a moderate clinical improvement (>37.5 decrease in weighted Pediatric CD Activity Index [wPCDAI] for CD or ≥30 decrease in Pediatric UC Activity Index [PUCAI] for UC) in comparison with their clinical score at diagnosis. This clinical improvement was lower at weeks 4, 12, and 24 in the group with low SLPI scores but became equal at week 52 (Supplemental Figure 4A). This clinical improvement may in part relate to the fact that, before treatment, patients with high SLPI scores tend to have higher clinical disease activity and therefore more chance to undergo improvement upon induction therapy. In contrast, fecal calprotectin remission (Fcal < 300 μg/g) followed a different course. While initially at 4 and 12 weeks there was similar Fcal remission between the groups, the proportion of patients with Fcal remission at weeks 24 and 52 was higher in the group of patients with low SLPI scores than in those with high scores (Supplemental Figure 4B). Together, these data may suggest that high intestinal SLPI at diagnosis does relate to a lower chance to reach Fcal remission at 1 year after diagnosis. However, as these patients were enrolled in a real-world data cohort with different kinds of induction and maintenance treatments, further data collection is needed to firmly establish such a relationship.

In conclusion, SLPI protein and mRNA expression is increased in the colonic epithelium of patients with CD or UC compared with non-IBD controls. Moreover, high colonic epithelial SLPI protein expression is associated with more severe clinical disease activity and macroscopic inflammation in both CD and UC.

### Colonic epithelial SLPI expression is associated with histological disease activity and infiltration of neutrophils.

**Figure 3:**
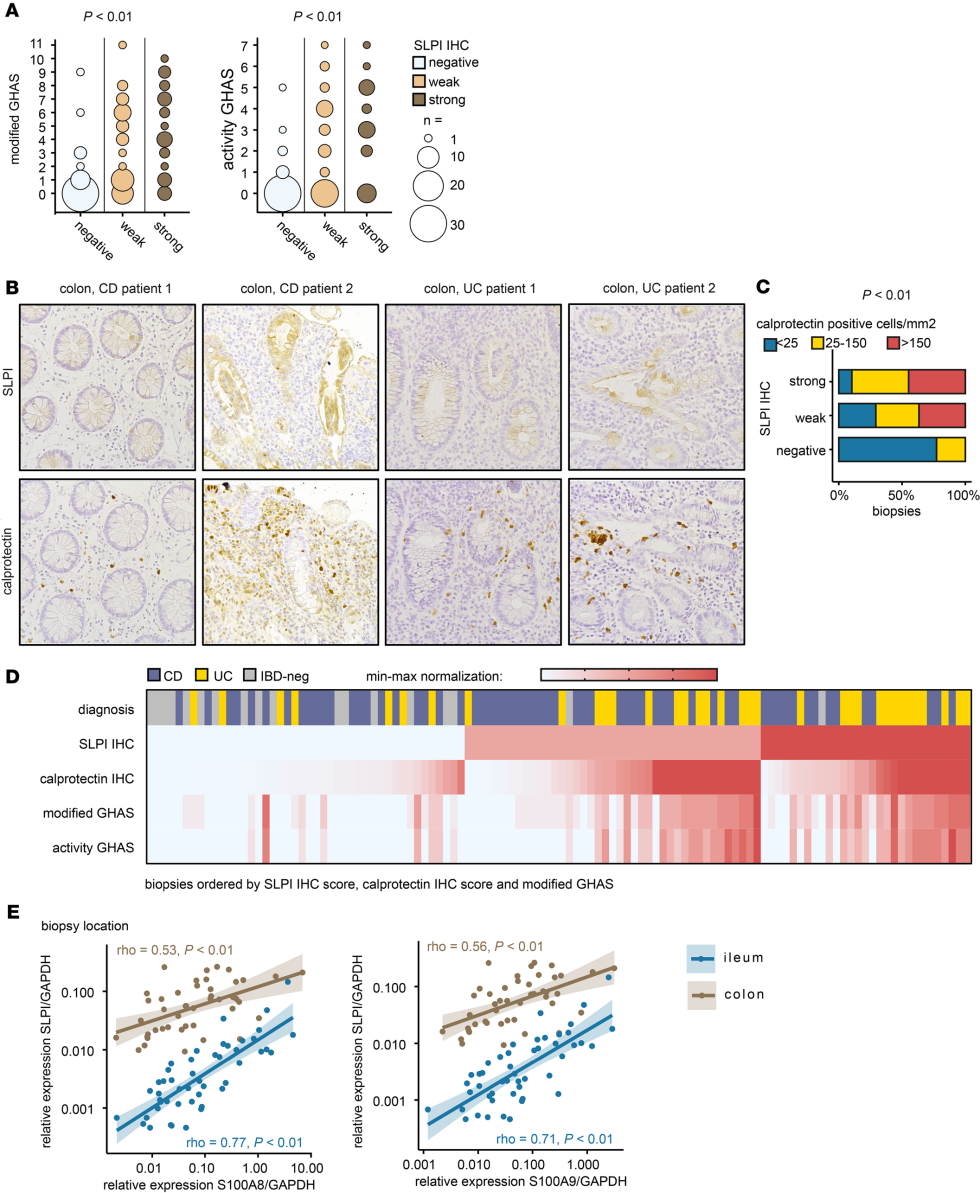
Colonic epithelial SLPI expression is associated with histological disease activity and with infiltration by calprotectin-positive cells. (**A**) H&E-stained sections of colonic biopsies from pediatric patients with CD (*n* = 41) or UC (*n* = 22) and IBD-negative (IBD-neg) controls (*n* = 15) were scored using the modified Global Histological Disease Activity Score (modified GHAS), including the subscore “activity GHAS.” GHAS scores are shown for the 3 categories of SLPI IHC scores. Kruskal-Wallis rank sum test. The size of the circles represents the number of biopsies (total *n* = 114 biopsies). (**B** and **C**) Calprotectin IHC in colonic biopsies from patients with CD or UC and IBD-negative controls. (**B**) Representative images of calprotectin IHC and SLPI IHC for colonic biopsies from 2 patients with CD and 2 patients with UC. Images were acquired at ×20 magnification. (**C**) The number of calprotectin-positive cells was counted per mm^2^ (maximum, 200) and grouped into 3 categories. Percentages of calprotectin scores per colonic SLPI IHC score (total *n* = 114 biopsies) are shown. Fisher’s exact test. For additional statistical comparisons, see Supplemental Table 2. (**D**) Heatmap of relationship between the different histological scores described above for all colonic biopsies (*n* = 114) from patients with CD (*n* = 40) or UC (*n* = 22) and IBD-negative controls (*n* = 15). Biopsies were ranked by SLPI IHC score, then by number of calprotectin-positive cells per mm^2^ (maximum, 200 “calprotectin IHC”), and then by modified GHAS. For each score, data are normalized to the minimum and maximum within that score. (**E**) *SLPI*, *S100A8*, and *S100A9* mRNA expression measured by qPCR in ileal and colonic biopsies from patients with CD (*n* = 21) or UC (*n* = 19) and IBD-negative controls (*n* = 9). Each data point represents one biopsy. Spearman’s rank correlation coefficient.

As epithelial SLPI expression was mainly increased in macroscopically inflamed regions of the colon in IBD, we next investigated which immune processes underlie this association. Microscopic disease activity (i.e., modified GHAS) was significantly associated with high SLPI scores in colonic biopsies from patients with CD or UC and IBD-negative controls (Kruskal-Wallis rank sum test, *P* < 0.01; Figure 3A). To assess whether microscopic disease activity is related to neutrophil infiltration, we assessed the relationship between SLPI and the activity GHAS, which reflects the presence of neutrophils in the lamina propria and epithelium, epithelial damage, and erosions or ulcers. Microscopic disease activity (activity GHAS) was significantly associated with high SLPI scores in colonic biopsies (Kruskal-Wallis rank sum test, *P* < 0.01; Figure 3A). The activity GHAS subscore “epithelial damage” positively related to SLPI expression (2-sided Fisher’s exact test, *P* < 0.01). Only a few biopsies showed erosion or ulcers (10/114). Of these, 8/10 showed weak/strong SLPI expression in the remaining epithelial cells versus 62/104 (60%) of the biopsies without erosion or ulcers (difference not statistically significant). To determine the relationship with neutrophil infiltration, we performed immunohistochemistry for calprotectin (S100A8/S100A9 heterodimer) on colonic biopsies from patients with CD or UC and IBD-negative patients.

Fecal calprotectin concentrations reflect the presence of neutrophils in the intestine and predict disease relapse in IBD (36). We observed clusters of calprotectin-positive cells in a subset of colonic biopsies (Figure 3B). The number of calprotectin-positive cells was significantly associated with the SLPI score (Fisher’s exact test, *P* < 0.01; Figure 3C). To visualize the relationships between SLPI expression detected by immunohistochemistry, the diagnosis, the number of calprotectin-positive cells, and microscopic disease activity, we generated a heatmap of the histological data from all colonic biopsies (Figure 3D). High colonic epithelial SLPI protein expression was associated with high numbers of calprotectin-positive cells and microscopically active disease.

To further assess the relationship between SLPI and calprotectin, we also measured *SLPI*, *S100A8*, and *S100A9* mRNA in ileal and colonic biopsies from patients with CD or UC and IBD-negative controls from the longitudinal IBD cohort. *SLPI* mRNA significantly correlated with *S100A8* and *S100A9* expression in both ileal biopsies and colonic biopsies (Figure 3E). Together, these data further demonstrate that colonic epithelial SLPI expression is associated with neutrophil infiltration.

### RNA sequencing reveals enrichment for immune activation and neutrophil and T cell infiltration pathways in the colon of patients with high colonic epithelial SLPI expression.

**Figure 4:**
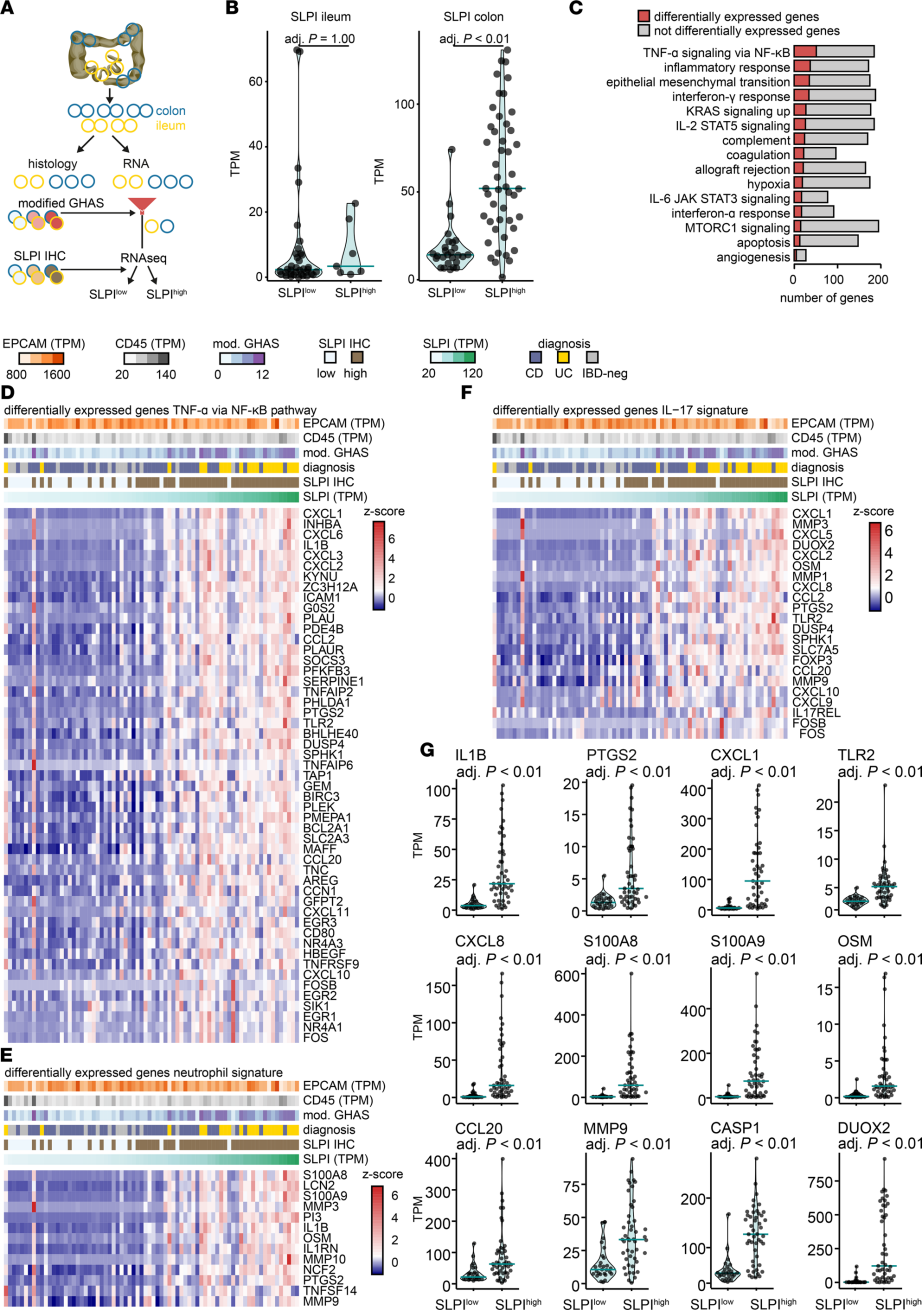
RNA sequencing reveals enrichment of immune activation and neutrophil and T cell infiltration pathways in paired colonic biopsies from patients with high SLPI IHC scores. (**A**) Two adjacent (paired) biopsies were collected from multiple ileal and colonic regions during endoscopy for therapy-naive patients with CD or UC and IBD-negative (IBD-neg) controls. One paired biopsy was used for histological analysis, and RNA sequencing was performed on the paired biopsy from the most affected ileal and colonic region per patient. The modified GHAS was used to define the most affected ileal and colonic region per patient. SLPI IHC scores of the most affected biopsy were used to classify patients as SLPI^low^ (negative IHC) or SLPI^high^ (weak/strong IHC). RNA sequencing was performed on the paired biopsy from the most affected ileal and colonic region per patient. (**B**–**F**) Intestinal gene expression of SLPI^high^ and SLPI^low^ groups; differentially expressed genes (DEGs) were defined as having an FDR-adjusted *P* value ≤ 0.05 and log_2_FC > 1 or < –1. (**B**) Transcripts per kilobase million (TPM) values for SLPI RNA in ileal and colonic biopsies from SLPI^low^ and SLPI^high^ groups. (**C**) Top 15 of the 26 upregulated hallmark pathways from gene set enrichment analysis. (**D**–**F**) *Z*-scored TPM values for DEGs in colonic biopsies from patients in the SLPI^high^ versus the SLPI^low^ group. Genes are ranked according to significance (lowest adjusted *P* value on top). Above the heatmaps, TPM values for EPCAM (as a measure of an epithelial signal) and CD45 (a hematopoietic cell signal), the dichotomized SLPI IHC score (SLPI^high^, SLPI^low^), the modified GHAS score from the paired biopsy, and the diagnosis are shown. (**D**) The 51 upregulated genes of 200 in the gene set “hallmark TNFA signaling via NFKB” pathway (M5890) (69, 70). (**E**) The upregulated gene set for the “neutrophil signature” contains known neutrophil-associated genes selected by us. (**F**) The upregulated gene set for the “IL-17 signature” was compiled using multiple pathways (see Supplemental Methods). (**G**) TPM values of representative DEGs (upregulated, adjusted *P* value ≤ 0.05 and log_2_FC > 1) in colonic biopsies from the SLPI^high^ versus the SLPI^low^ group.

Neutrophils are a heterogeneous cell population with highly varied immune activity; thus, we investigated whether neutrophils that colocalize with high epithelial SLPI expression exhibit particular immune profiles, and examined the cellular networks these cells may cooperate with. We conducted RNA sequencing of paired biopsies from the same patients for whom we performed SLPI immunohistochemistry. One ileal and one colonic biopsy corresponding to the location of the microscopically most affected ileal and colonic region were included for each patient (Figure 4A). Considering the statistical requirement of defining 2 groups for the differential expression analysis, we categorized patients into SLPI-high (SLPI^high^; weak/strong IHC) and SLPI-low (SLPI^low^; negative IHC) epithelial protein expression groups according to the SLPI immunohistochemistry scores for their most affected colonic and ileal biopsies (Figure 4A).

Based on ileal SLPI protein expression, only 7 patients were categorized into the SLPI^high^ group versus 36 into the SLPI^low^ group (Figure 4B). As anticipated, *SLPI* mRNA expression was not different between the SLPI^high^ and SLPI^low^ groups, because only a small proportion of the ileal biopsies exhibited increased *SLPI* mRNA expression (Figure 4B). In contrast, in colonic biopsies, *SLPI* mRNA expression was significantly higher in the SLPI^high^ group compared with the SLPI^low^ group (based on colonic SLPI protein expression; Figure 4B). In the colon, 559 genes were differentially expressed between the SLPI^high^ and SLPI^low^ groups (487 upregulated, 72 downregulated); in the ileum, only 5 genes were differentially expressed between these groups (all upregulated; Wald test with Benjamini-Hochberg correction: log_2_ fold change [log_2_FC] < –1 or > 1 and adjusted *P* value < 0.05). These data demonstrate that histological detection of SLPI protein expression in colon discriminates 2 groups of IBD patients with distinct gene expression profiles in the most affected colonic region. Therefore, we focused on colonic SLPI expression in our subsequent analyses. In support of the observation that SLPI is mainly expressed by goblet cells, expression of mucin genes was higher in colonic biopsies from the SLPI^high^ group than in those from the SLPI^low^ group (Supplemental Figure 5).

To identify which pathways are associated with high colonic SLPI expression, we performed gene set enrichment analysis (GSEA) of the expression profiles from SLPI^high^ and SLPI^low^ colonic biopsies. Significant enrichment of 26/50 hallmark pathways was observed in SLPI^high^ patients. The top 15 upregulated pathways were associated with multiple immune processes including TNF-α signaling via NF-κB, the epithelial-mesenchymal transition, and interferon-γ (IFN-γ) responses (Figure 4, C–F). The most highly upregulated genes in the “TNF-α signaling via NF-κB” hallmark pathway were the neutrophil-attracting chemokines *CXCL1*, *CXCL3*, and *CXCL6*, the inflammatory cytokine *IL1B*, the pattern recognition receptor Toll-like receptor 2 (*TLR2*), and the prostaglandin synthesis gene cyclooxygenase-2 (*PTGS2*) (Figure 4, D and G). In keeping with the strong neutrophil infiltration observed on histology, biopsies from SLPI^high^ patients exhibited a signature of not only neutrophil infiltration, but also functional activation — as indicated by enrichment of *OSM* (involved in stromal cell activation), matrix metalloproteinase 9 (*MMP9*; involved in collagen degradation), the antimicrobial proteins calprotectin (*S100A8*/*S100A9* heterodimer) and lipocalin-2 (*LCN2*), and neutrophil cytosolic factor 2 (*NCF2*; encodes a subunit of the NADPH complex required for microbial killing) (Figure 4, E and G). These findings were supported by upregulation of multiple genes in the GSEA-curated “neutrophil degranulation pathway” (37) in the SLPI^high^ group (Supplemental Figure 6A).

In addition to neutrophil activation pathways, colonic biopsies from the SLPI^high^ group versus the SLPI^low^ group were enriched in IL-17 signaling (Figure 4, F and G). Key upregulated genes included the chemokine *CCL20*, which attracts CCR6-expressing Th17 and ex-Th17 cells to the inflamed intestine (38); *CXCL8*, a hallmark neutrophil-recruiting chemokine induced by IL-17 signaling; and epithelial antimicrobial dual oxidase 2 (*DUOX2*) (Figure 4, F and G). Elevated ileal *DUOX2* expression has been associated with expansion of proteobacteria in both CD and UC, which may possibly be related to microbial shifts in the SLPI^high^ subgroup (39).

Most strikingly, an overall signature of genes previously associated with resistance to therapy was detected in the SLPI^high^ group (10, 11). Thus, we analyzed whether previously described expression modules related to resistance to therapy were enriched in SLPI^high^ patients (10, 11). Indeed, most genes from the OSM-associated module (10), which differentiates patients with primary non-responsiveness to anti-TNF therapy, were upregulated in colonic biopsies from our therapy-naive SLPI^high^ IBD patients (Supplemental Figure 6B). Intriguingly, caspase-1 (*CASP1*), the factor required for activation of IL-1B — and likely an important driver of inflammation in this module — was highly upregulated in SLPI^high^ patients (Figure 4G).

Moreover, a previously described neutrophil module associated with non-response to therapy (11) was upregulated in colonic biopsies from the SLPI^high^ group (Supplemental Figure 6C). To further strengthen our claim that epithelial SLPI protein expression is a biologically relevant parameter relating to underlying immune disease, we performed unbiased hierarchical clustering of patients using differentially expressed genes identified by comparison of colonic biopsies of CD versus IBD-negative and UC versus IBD-negative patients. Subsequent Ward’s hierarchical clustering on the basis of these differential transcriptional profiles in colonic biopsies, indeed, clustered CD and UC patients into groups with high or low SLPI protein expression on immunohistochemistry (Supplemental Figure 7).

Overall, these results indicate that colonic epithelial SLPI expression is associated with strong immune activation, characterized by neutrophil activation and IL-17 signaling in IBD. In addition, genes associated with non-response to therapy were enriched in the colon of IBD patients with high epithelial SLPI expression.

### IL-17A production is increased in the colon and peripheral blood of patients with high colonic epithelial SLPI expression.

**Figure 5:**
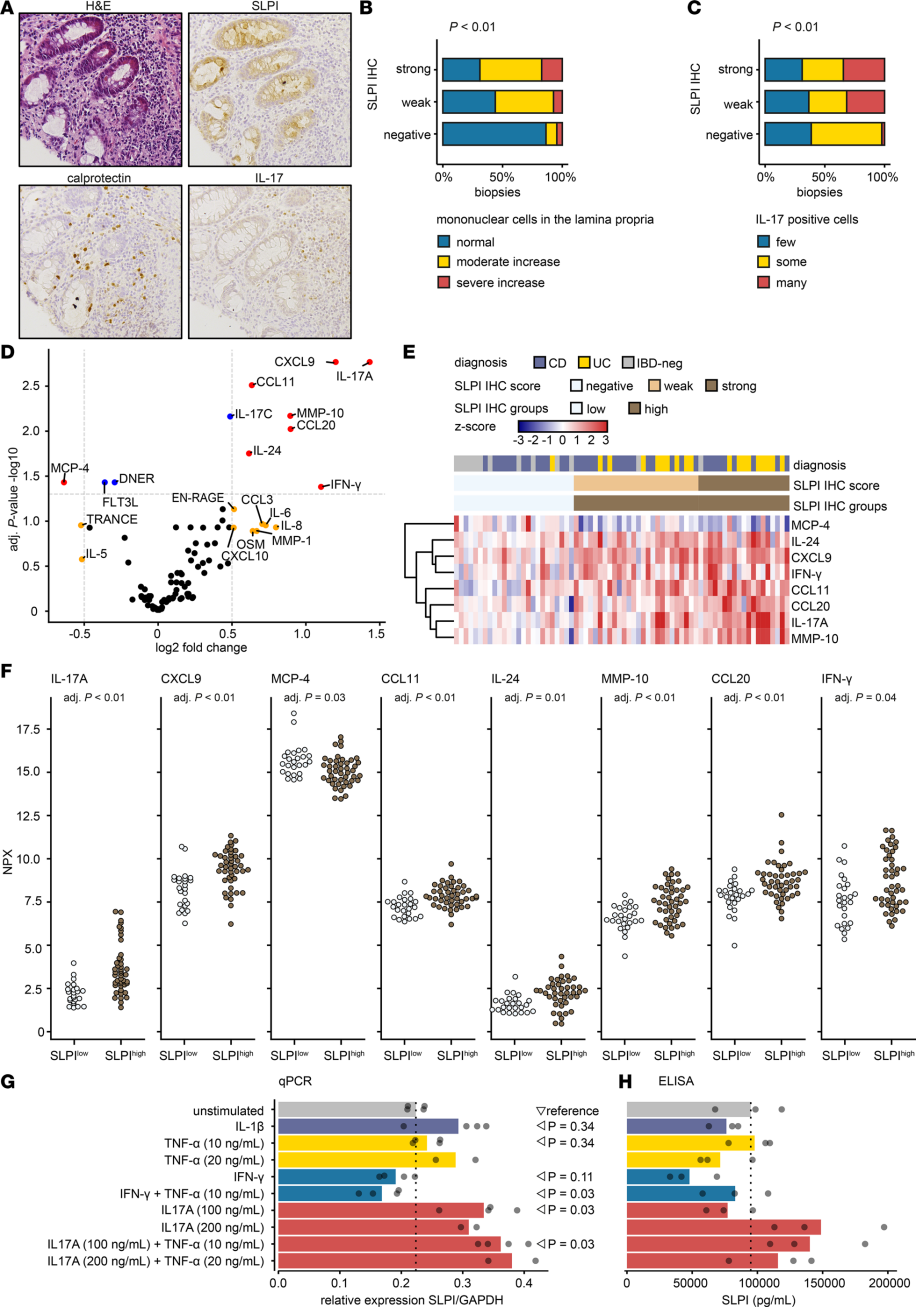
Colonic epithelial SLPI protein expression is associated with IL-17–positive cells in the tissue and IL-17A production in peripheral blood. (**A**) H&E staining and IHC for SLPI, calprotectin, and IL-17 on colonic biopsies from patients with CD (*n* = 41) or UC (*n* = 22) and IBD-negative (IBD-neg) controls (*n* = 15). Representative images acquired at ×20 magnification. (**B**) The GHAS parameter “infiltration by mononuclear cells in the lamina propria” scored on H&E-stained sections for the 3 categories of SLPI IHC scores (total *n* = 114 biopsies). Fisher’s exact test. (**C**) The number of IL-17–positive cells in the lamina propria was classified into 3 categories and plotted for the 3 categories of SLPI IHC scores (total *n* = 114 biopsies). Fisher’s exact test. For additional statistical comparisons, see Supplemental Table 2. (**D**–**F**) SLPI IHC scores were used to classify patients as SLPI^low^ (negative IHC, *n* = 25) or SLPI^high^ (weak/strong IHC, *n* = 45). Protein concentrations in plasma were compared between the 2 groups (2-tailed independent *t* test; Benjamini-Hochberg correction). (**D**) Differentially abundant proteins (log_2_FC < –0.5 or > 0.5, adjusted *P* value < 0.05) between the SLPI^high^ and SLPI^low^ groups are shown in red. (**E**) *Z* scores of the normalized protein expression (NPX) values of increased proteins in IBD; zero represents the NPX of non-IBD control. Ward’s clustering criterion (67, 68). (**F**) NPX values for differentially abundant proteins (log_2_FC < –0.5 or > 0.5, adjusted *P* value < 0.05) in the SLPI^high^ compared with the SLPI^low^ group. (**G** and **H**) TR146 buccal epithelial cells were cultured and serum-starved for 4–24 hours (**G**) or 24 hours (**H**) and subsequently stimulated with IL-1β, TNF-α, IFN-γ, or IL-17A or unstimulated for 16 hours. (**G**) Relative expression of SLPI mRNA compared between unstimulated and stimulated cells measured by qPCR. (**H**) Concentrations of SLPI protein in the supernatant measured by ELISA. (**G** and **H**) Bars represent the mean of multiple culture wells. Wilcoxon’s rank sum test.

As we observed enrichment of IL-17 signaling pathways in colonic biopsies from patients with high epithelial SLPI protein expression (SLPI^high^), we questioned whether Th17 lymphocytes were increased in these biopsies. First, we estimated the number of mononuclear cells in the lamina propria of colonic biopsies from patients with CD or UC and IBD-negative controls. The number of mononuclear cells in the lamina propria was higher in SLPI^high^ colonic biopsies (Fisher’s exact test, *P* < 0.01; Figure 5, A and B). As these mononuclear cells could be monocytes, macrophages, or lymphocytes, we performed immunohistochemistry to detect IL-17–expressing lymphocytes. IL-17–positive cells were increased in a subset of biopsies, especially in SLPI^high^ biopsies with high numbers of calprotectin-positive cells (Figure 5, A and C). The number of IL-17–positive cells was significantly associated with colonic epithelial SLPI protein expression (Fisher’s exact test, *P* < 0.01; Figure 5C).

To further explore the association between colonic epithelial SLPI protein expression and immune activation, we measured protein concentrations in plasma from patients with CD or UC and IBD-negative controls. Strikingly, the plasma concentrations of the Th17-associated cytokines IL-17A, CCL20, and IL-24 were significantly elevated in the SLPI^high^ group versus the SLPI^low^ group (Figure 5, D–F), indicating that high colonic SLPI expression is associated with a Th17-like immune protein profile in plasma. IL-24 is produced by Th17 lymphocytes in response to IL-17A, via autocrine negative feedback (40). Notably, IFN-γ plasma protein concentrations were also significantly increased in the SLPI^high^ group, but at a lower fold change compared with IL-17A, suggesting a stronger association between epithelial SLPI expression and a Th17 response (Figure 5, D–F). These data indicate that high colonic epithelial SLPI expression is associated with elevated Th17 in the peripheral blood in IBD.

As IL-17A enhances SLPI expression in human lung epithelial cells (41), we questioned whether IL-17A upregulates SLPI in the intestinal epithelium. Previously, we have demonstrated that modulation of SLPI expression can best be modeled in the buccal epithelial cell line TR146, while the colonic epithelial cell line Caco-2 is more heterogeneous and varies in its capacity to express SLPI (13). We stimulated TR146 human buccal epithelial cells with IL-17A and other cytokines to induce SLPI in these cells (13). As expected, IFN-γ significantly downregulated *SLPI* (Figure 5G). Interestingly, IL-17A significantly upregulated *SLPI* mRNA (Figure 5G) and SLPI protein in the supernatant (Figure 5H), which suggests that colonic epithelial SLPI protein expression in IBD may in part occur as a result of elevated levels of IL-17A. As IL-17A also promotes tissue recruitment of neutrophils (42), our data suggest that IL-17A enhances both epithelial SLPI expression and accumulation of neutrophils in the colon in IBD.

### Circulating neutrophils from SLPI^high^ and SLPI^low^ patients have intrinsically different transcriptomes.

**Figure 6:**
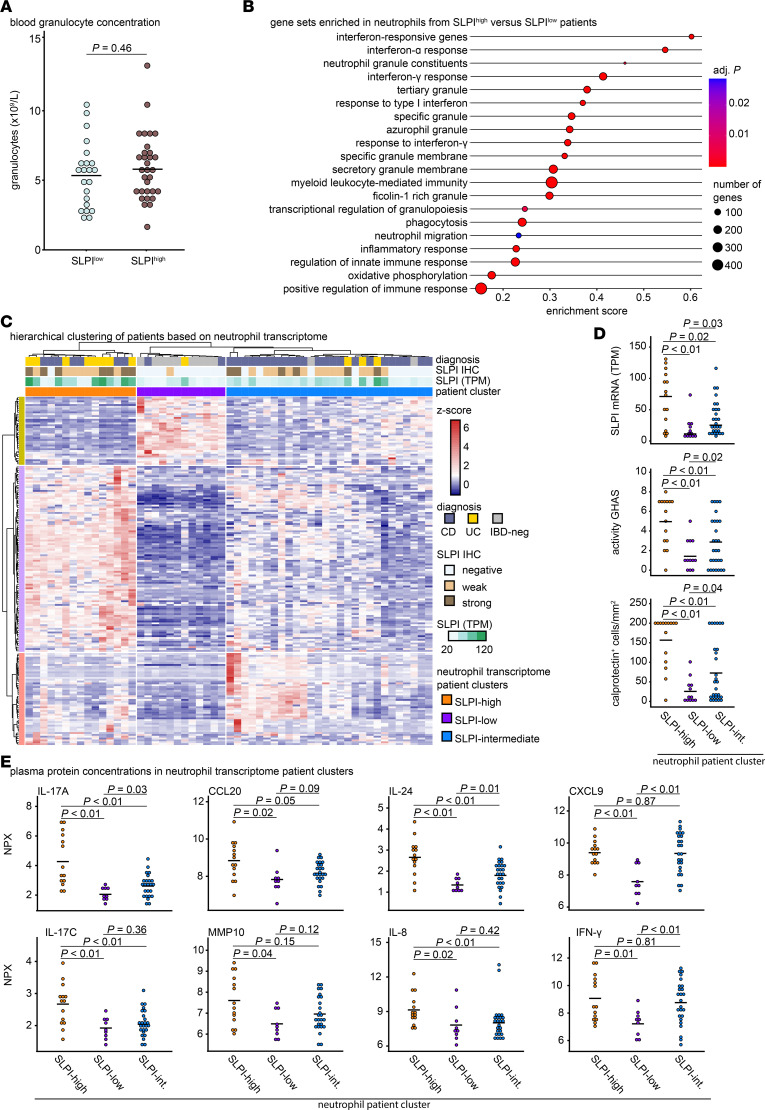
Circulating neutrophils from SLPI^high^ and SLPI^low^ patients have intrinsically different transcriptomes. (**A**) Granulocyte concentrations in peripheral blood from SLPI^high^ versus SLPI^low^ patients (based on SLPI IHC scores). Bars represent the mean; 2-tailed Student’s *t* test. (**B**) Gene set enrichment analysis performed on the neutrophil transcriptome from SLPI^high^ versus SLPI^low^ patients and significant (adjusted *P* value < 0.05) gene sets from the curated C2, C5, and hallmark collections. The size of the circles represents the total number of genes in the gene set. (**C**) Heatmap of *z*-scored TPM values for all 188 DEGs in circulating neutrophils from SLPI^high^ patients compared with SLPI^low^ patients. Hierarchical clustering of patients on the basis of *z*-scored TPM values for DEGs in neutrophils with Ward linkage (48, 49). Three neutrophil transcriptome patient clusters were identified: SLPI-high, SLPI-low, and SLPI-intermediate. (**D**) *SLPI* mRNA expression, activity GHAS, and number of calprotectin-positive cells in the paired colonic biopsies of patients categorized based on the neutrophil transcriptional clusters identified in the heatmap (**C**); bars represent the mean. Wilcoxon’s rank sum test. (**E**) Plasma protein concentration (normalized protein expression [NPX]) values across unbiased patient clusters identified by neutrophil transcriptome in the heatmap (**C**). (**E**) Bars represent the mean. Wilcoxon’s rank sum test.

Since the colonic epithelial SLPI^high^ immunotype was associated with strong immune activation and systemic IL-17A–related immune protein profiles, we questioned whether circulating neutrophils are differentially programmed in SLPI^high^ and SLPI^low^ patients before migrating into the tissue. We performed bulk RNA sequencing of freshly isolated peripheral blood neutrophils at diagnosis. The total concentrations of neutrophils were not significantly different between the patient groups, excluding large changes in granulopoiesis (Figure 6A).

Analysis of the neutrophil transcriptomes identified 188 differentially expressed genes (DEGs) between SLPI^high^ and SLPI^low^ patients. GSEA revealed that neutrophils from SLPI^high^ patients were significantly enriched in multiple functional pathways, including neutrophil granule formation, phagocytosis, oxidative phosphorylation, and interferon signaling (Figure 6B). Hierarchical clustering on the basis of all DEGs in neutrophils yielded 3 distinct patient clusters. Strikingly, despite the fact that we clustered based on mRNA expression in circulating neutrophils, this clustering differentiated patients with different degrees of SLPI mRNA and protein expression in their most affected colonic biopsy. Therefore, we named these neutrophil clusters SLPI-high, SLPI-intermediate, and SLPI-low (Figure 6, C and D, and Supplemental Figure 8), and the key transcriptional features driving the definition of these clusters are listed in Supplemental Table 4. These patient clusters based on neutrophil expression patterns were associated with differences in histological disease severity and neutrophil infiltration in the paired colonic biopsies (Figure 6D). This demonstrates that patients with high intestinal SLPI expression (Supplemental Figure 8) and associated intestinal disease exhibit altered molecular programming of immune function in circulating neutrophils. In line with this systemic alteration, circulating inflammatory protein concentrations differed significantly between the 3 neutrophil transcription–based patient clusters (Figure 6E). Both the SLPI-intermediate and -high clusters had higher plasma CXCL9, IL-24, and IFN-γ concentrations compared with the SLPI-low cluster (Figure 6E).

Crucially, clustering on the basis of the neutrophil transcriptome also identified patients with increased plasma IL-17A and IL-17C, particularly in the SLPI-high cluster (Figure 6E).

Overall, these results demonstrate that high epithelial SLPI expression and concomitant extensive neutrophil infiltration, IL-17A production, and histological damage collectively identify a subgroup of patients with IBD that exhibit altered molecular programming of circulating neutrophils.

## Discussion

IBD is a heterogeneous disease, and identification of distinct disease subtypes is needed to improve therapeutic responses. We anticipated that histological characterization of intestinal tissue from patients with IBD could indicate distinct underlying immune processes. Here, we demonstrate that immunohistochemical detection of high SLPI protein expression in epithelial cells at diagnosis identifies a subgroup of patients with extensive inflammatory neutrophil infiltration, increased IL-17A production, increased intestinal permeability, and high clinical disease activity. Crucially, circulating neutrophils from SLPI^high^ patients are intrinsically different and exhibit altered transcription of genes involved in neutrophil granule formation, phagocytosis, oxidative phosphorylation, and interferon signaling — which may consequently alter microbial-host interactions and tissue damage (43).

SLPI is a very diverse protein that prevents tissue damage by inhibiting proteases, including neutrophil elastase (16). SLPI also exerts antimicrobial activity and can inhibit NF-κB–mediated inflammatory gene transcription (17–19). However, the main reason that prompted us to assess SLPI expression as a histological parameter in IBD is that repetitive microbial interactions upregulate SLPI expression in the epithelium (13). We demonstrated that increased microbial pressure upon breach of barrier integrity further increased colonic SLPI expression in 2 murine models of colitis. Moreover, in silico analyses of intestinal mRNA data demonstrated increased colonic *SLPI* expression in 2 cohorts of adults with IBD who had received treatment versus healthy controls. Thus, we questioned whether quantification of SLPI protein by histology would reflect a disturbed microbial host immune interaction in our 2 independent cohorts of therapy-naive pediatric patients with IBD. We found that SLPI is produced mainly by colonic epithelial goblet cells in macroscopically inflamed regions of the colon and — to a lesser extent — in the ileum. This indicates that upregulation of SLPI reflects a biological process related to the ongoing inflammation in IBD. Indeed, the intensity of epithelial SLPI expression was associated with the severity of microscopic disease activity in colonic biopsies from the pediatric patients with IBD. On histology, SLPI expression was related to epithelial damage and was not lost in the remaining epithelial cells in the few biopsies with erosion and/or ulcers. In particular, our data argue that infiltration by calprotectin-positive neutrophils is related to high epithelial SLPI expression in the colon. As neutrophils contain relatively little mRNA and are vulnerable to cell death, neutrophil signaling in IBD tissues is difficult to assess. However, recent studies indicated that high neutrophil infiltration is associated with non-response to treatment in IBD (11). Although intestinal macrophages can express SLPI (44), we observed very few SLPI-positive cells in the lamina propria in biopsies from IBD patients and therefore could not establish whether SLPI expression in macrophages also increases in IBD (data not shown). Overall, our scoring of histological disease activity and immunohistochemical analysis identified a relationship between high epithelial SLPI expression and neutrophil infiltration in pediatric IBD.

High SLPI expression has not previously been reported in IBD. As dense infiltration by neutrophils is associated with epithelial cell loss in IBD, upregulation of SLPI may have been overlooked in previous bulk RNA analyses. However, our immunohistochemical analysis accurately discriminated 2 groups of patients with high or low SLPI expression in epithelial cells.

Crucially, high epithelial SLPI expression was observed in the most affected colonic biopsy from a subgroup of patients with IBD with high clinical disease activity, which demonstrates that colonic SLPI expression can indicate disease activity at the patient level. It is important to note that in both mice and humans, SLPI expression predominates in the colon compared with small intestine (13). It is unresolved whether this depends on the presence of particular microbial species or whether this relates to a particular type of epithelial (precursor) cell. In our study, SLPI expression was most detected in goblet cells, which are indeed less numerous in the small intestine. In line with this, increased intestinal SLPI expression in IBD most prominently occurs in the colon. In consequence, care should be taken in examining CD patients with isolated ileal disease. Overall, our data demonstrate that high colonic epithelial SLPI expression is a component of a strong antimicrobial response that is not present in all patients with IBD at diagnosis, which suggested that the SLPI^high^ subgroup may have distinct underlying immune dysfunction.

In line with this suggestion, RNA sequencing revealed a particular pattern of immune activation related to high colonic epithelial SLPI expression and neutrophil infiltration. GSEA analyses revealed strong enrichment of a variety of inflammatory immune pathways involved in both innate and adaptive immune responses in biopsies with high SLPI expression. The most enriched genes were involved in neutrophil recruitment, tissue remodeling, host-microbiota interactions, and T cell infiltration. Strikingly, genes associated with resistance to therapy — including *OSM*, a neutrophil product, and its associated module (10, 11) — were among the most upregulated genes. In addition, genes in the IL-1 stromal-neutrophil interaction network, previously associated with the failure of therapy, were significantly increased (11). Most importantly, intrinsic differences in neutrophil function were observed between SLPI^high^ and SLPI^low^ patients, as circulating neutrophils from SLPI^high^ patients exhibited transcriptomic changes in granule formation, phagocytosis, oxidative phosphorylation, and interferon signaling — prior to these cells entering the intestinal tissue. These data argue that, in characterizing heterogeneity of IBD lesions, not only the number of infiltrating neutrophils may be relevant, but more so their (transcriptional) programming, which strongly relates to the degree of epithelial SLPI expression in the intestine.

High colonic epithelial SLPI expression was also associated with IL-17 signaling, increased IL-17–positive cells in the colonic lamina propria, and a Th17-like immune protein profile in peripheral blood. As Th17 lymphocyte differentiation is induced by microbial-host interactions (45), IL-17 signaling and epithelial upregulation of SLPI are likely induced via the same antimicrobial response. In fact, we confirmed that IL-17A enhanced SLPI mRNA and protein expression in an epithelial cell line, which indicates that SLPI expression can be upregulated both directly by microbial contact and indirectly via IL-17A production. This is similar to the regulation of SLPI in airway epithelium; in a murine model, SLPI expression was upregulated by IL-17A after colonization with *Bordetella pseudohinzii*, a murine-adapted airway microbe, which conferred protection from lung inflammation (41). Therefore, we hypothesize that high SLPI expression in the colonic epithelium reflects an antimicrobial response to microbiota interaction that occurs in a subgroup of patients with IBD. It remains to be determined whether epithelial SLPI expression in the colon is beneficial or detrimental. As SLPI has been reported to play a role in wound healing (31) and may protect mucosal tissues against inflammation by inhibiting proteases, suppressing chemokine production, and killing microorganisms (20–23), we expect that increased SLPI production could limit intestinal tissue damage and promote tissue healing.

Overall, this study demonstrates that colonic epithelial SLPI expression occurs in a subgroup of patients with IBD with a distinct immune response and high clinical disease activity at diagnosis. Our observation has relevance as immunohistochemical detection of SLPI is reliable and simple, and semiquantitative scoring of SLPI protein strongly correlates with SLPI mRNA expression and could add to deciphering of various patient immunotypes. As such, the information provided by the semiquantitative SLPI score could be used independent of the more complex multiparameter GHAS or in conjunction to obtain more insight into the patient’s underlying immune disease.

## Methods

### Sex as a biological variable.

In all human and murine experiments, males and females were included.

### PIBD-SETQuality subcohort.

As part of the PIBD-SETQuality, patients less than 18 years old with suspected IBD were included at diagnostic endoscopy (34) and diagnosed according to the revised Porto criteria (46). Participants with intestinal complaints and clinical suspicion of IBD who did not receive the IBD diagnosis after endoscopy were included as IBD-negative controls (IBD-neg).

Biopsies collected at diagnostic endoscopy at the Erasmus Medical Centre were available for a subcohort of 78 patients. No patients received treatment for IBD before diagnostic endoscopy. All patients and parents signed informed consent for collection of biomaterials.

Endoscopic disease was scored according to the Simple Endoscopic Score for Crohn’s Disease (SES-CD) (47) for CD and the UC Endoscopic Index of Severity (UCEIS) (48) for UC. As the original UCEIS is determined by the score of the most inflamed segment only (range, 0 to 8), we also summed the UCEIS for each colonic segment to obtain a score representing the whole colon (range, 0 to 40). SES-CD and total UCEIS were only analyzed for patients with complete endoscopy (terminal ileum to rectum). Disease activity was scored using the weighted Pediatric CD Activity Index (wPCDAI) (49) for CD and the Pediatric UC Activity Index (PUCAI) (50) for UC. Scores were categorized as “none” (wPCDAI < 12.5; PUCAI = 0), “mild” (wPCDAI 12.5–40; PUCAI 10–30), “moderate” (wPCDAI > 40–57.5; PUCAI 42.5–60), and “severe” (wPCDAI > 57.5; PUCAI 70–80) using validated cutoffs. Disease location, disease behavior, and disease extent were scored using the Paris classification (51).

Biopsies were collected from macroscopically non-affected and macroscopically affected ileum and colon. Adjacent “paired” biopsies were collected from each location for histological analysis and RNA sequencing. Biopsies for histological analysis were fixed by incubation in 4% paraformaldehyde for 4 hours at room temperature, and thereafter overnight at 4°C, then stored in 70% ethanol at 4°C (<12 hours to >3 weeks) and paraffin-embedded, and 4-μm-thick sections were mounted on Polysine adhesion glass slides (Thermo Fisher Scientific). Biopsies for RNA sequencing were placed in RNA*later* tissue storage reagent (Sigma-Aldrich) overnight at 4°C, then stored at –80°C.

EDTA blood was collected and centrifuged at 321*g* for 10 minutes at room temperature. Plasma was collected and stored at –80°C.

### Longitudinal pediatric IBD cohort.

Patients less than 18 years old with suspected IBD were included at diagnostic endoscopy from 2007 until 2020 and diagnosed according to the revised Porto criteria (46). Biopsies were collected from both macroscopically non-affected and macroscopically affected segments of the ileum and colon, during diagnostic endoscopy at the Erasmus Medical Centre, and stored as described above. No patients were treated for IBD before diagnosis. All patients and parents signed informed consent for collection of biomaterials.

### Muc2-deficient mice.

Mucin 2–deficient (*Muc2*-deficient) mice (29) were cohoused with wild-type littermates. Distal colonic tissue was collected for histological analysis, fixed in 4% paraformaldehyde, and paraffin-embedded, as described previously (29). Five-μm-thick sections were mounted on Polysine adhesion glass slides (Thermo Fisher Scientific). RNA extraction from distal colonic tissue and cDNA synthesis for quantitative PCR were conducted as described previously (29).

### DSS-induced colitis.

DSS-induced colitis is described in Supplemental Methods. Briefly, a total of 11 C57BL/6 wild-type mice received 2% DSS in drinking water ad libitum for 5 days in 2 independent experiments; 3 C57BL/6 wild-type littermates were untreated. Fecal samples were collected daily from day 1 until day 10. Mice were sacrificed on day 5, 10, or 36 (32).

### Culture of TR146 cells.

TR146 buccal epithelial cells (13), originally provided by Mark Herzberg, University of Minnesota, Minneapolis, Minnesota, USA were cultured in DMEM and stimulated with recombinant human IL-1β (Immunotools, 11340013), recombinant human TNF-α (BD Biosciences, 554618), recombinant human IFN-γ (Immunotools, 11343536), or recombinant human IL-17A (BioLegend, 570502) for 16 hours (concentrations indicated in figure legends), after which supernatants were collected and stored at –20°C to measure SLPI protein. Quantitative PCR (qPCR) and enzyme-linked immunosorbent assay (ELISA) were used to measure SLPI mRNA expression in cells and protein in supernatant, respectively (see Supplemental Methods).

### Measurement of SLPI in murine fecal samples.

Murine SLPI in fecal samples was measured using an ELISA, following the protocol described for human SLPI above, except the plates were coated with 0.2 μg/mL polyclonal antibody against mouse SLPI (R&D Systems/Bio-Techne, AF1735) in bicarbonate buffer (pH 9.5) overnight at 4°C. Recombinant mouse SLPI (MyBioSource, BMS2012482) was used to generate standard curves (up to 250 ng/μL). Murine SLPI was detected with 0.4 μg/mL biotinylated polyclonal antibody against mouse SLPI (R&D Systems/Bio-Techne, BAF1735).

### Immunohistochemistry.

H&E staining is described in Supplemental Methods. For immunohistochemistry, sections were deparaffinized in xylene, rehydrated in ethanol, and incubated in 3% H_2_O_2_ in PBS for 20 minutes to quench endogenous peroxidase activity. Antigen retrieval was performed by microwave treatment in citrate buffer (10 mM, pH 6.0) for human tissues and incubation with 0.1% pepsin from porcine gastric mucosa (Sigma-Aldrich, P7000) in 0.01 M HCl for 7 minutes at 37°C for murine tissues. Sections were blocked for 1 hour at room temperature in Tris buffer (10 mM, pH 8.0) containing 5 mM EDTA (pH 8.0), 0.15 M NaCl, 0.25% gelatin, 0.05% Tween 20, and 10% normal human serum (AB serum, Sanquin) for human tissue or 10% normal mouse serum (Thermo Fisher Scientific) for murine tissue, plus 10% serum matching the species in which the secondary antibody was raised (Supplemental Table 1). Sections were stained with primary antibody in PBS overnight at 4°C followed by biotinylated secondary antibody for 1 hour at room temperature, developed using Vectastain ABC Elite Kit (Vector Laboratories) and 3,3′-diaminobenzidine tetrahydrochloride (Sigma-Aldrich), counterstained with hematoxylin (Vector Laboratories), dehydrated, immersed in xylene, and mounted in Entallan (Sigma-Aldrich). Images were acquired using a Leica DM5500B microscope and Leica DFC450 C camera and analyzed using Leica Application Suite X software (Leica Microsystems). Sera and antibodies used for immunohistochemistry are listed in Supplemental Table 1.

### Scoring of human epithelial SLPI protein intensity.

The intensity of SLPI protein expression in the cytoplasm of epithelial cells was manually scored for each biopsy in a semiquantitative manner as “weak,” “moderate,” or “strong.” Scoring was designed separately for the monoclonal and polyclonal SLPI antibodies. The observer was blinded to clinical information. The scoring strategy was based on the range of SLPI staining intensity observed across all intestinal biopsies. Reproducibility of SLPI scoring with the monoclonal antibody was previously demonstrated in colorectal cancer (*n* = 507 patients) (52). A second pathologist independently scored tissue microarrays, and the linear weighted κ value was 0.62, indicating a fair to good interobserver agreement (52). In this study, semiquantitative SLPI scoring strongly related to SLPI mRNA expression in paired colonic biopsies, supporting the reliability of the scoring (Supplemental Figure 1).

### Scoring of global histological disease activity.

H&E-stained biopsies from the PIBD-SETQuality subcohort were scored by an experienced gastrointestinal pathologist using a modification of the Global Histological Disease Activity Score (GHAS) (53); the parameter “presence of granulomas” was excluded because biopsies from patients with UC were included, and “number of biopsies affected” was excluded because at most 2 biopsies were taken from the same anatomic region, according to a modification of the GHAS described by Li et al. (54). For the “activity GHAS” subscore, the parameters “architectural changes” and “infiltration of mononuclear cells in lamina propria” were excluded to study disease activity (versus chronic changes) (54).

The most affected (highest modified GHAS) ileal biopsy and colonic biopsy were chosen for each patient. If multiple biopsies had the same modified GHAS, a biopsy from a macroscopically inflamed region was chosen over a biopsy from a macroscopically non-inflamed region. Observers were blinded to clinical information.

### Olink proximity extension assay.

Multiplex proximity extension assay (PEA) using the Olink Target 96 Inflammation panel (95302) was performed on patient plasma samples by Olink Proteomics (55). This antibody-based immunoassay merged with qPCR detection quantifies multiple proteins simultaneously. Data are expressed as normalized protein expression values, an arbitrary unit on log_2_ scale acquired by normalization of qPCR values that reflects relative protein abundance.

### RNA sequencing of biopsies.

One ileal and one colonic biopsy were selected per patient based on the highest modified GHAS for the paired biopsy, as described above. RNA preparation is described in Supplemental Methods. Briefly, library preparation was performed on 500 ng total RNA using the KAPA mRNA HyperPrep Kit (Roche). The concentration and size distribution of the libraries were measured using the Qubit dsDNA HS Assay (Thermo Fisher Scientific) and High Sensitivity DNA Kit for BioAnalyzer (Agilent), respectively. Libraries were diluted to 2 nM and paired-end-sequenced on a NovaSeq 6000 (Illumina) with 2 × 100 read length at an expected library size of approximately 40 million reads per sample.

### RNA sequencing of neutrophils.

Neutrophils were isolated from 500 μL whole blood using the EasySep Direct Human Neutrophil Isolation Kit (STEMCELL Technologies Germany). Sample processing and library preparation are described in Supplemental Methods. Briefly, cDNA was generated from neutrophil lysates with the NucleoSpin RNA XS extraction kit (Macherey-Nagel) and SensiFAST cDNA synthesis kit. Libraries were prepared with the ssDNA Library Prep Kit (Integrated DNA Technologies), followed by a post-library ribodepletion (SEQuoia RiboDepletion Kit, Bio-Rad). Concentrations and library size distribution were determined before proceeding to the NovaSeq 6000 as described in *RNA sequencing of biopsies* above.

### Statistics.

Statistical analyses and visualization were performed using R version 3.5.1 (56), except for Olink PEA data analysis (see below). R package ggplot2 (57) was used for visualization; vipor (58) and ggbeeswarm (59) to generate violin plots (all violins had the same area before tail trimming); and pheatmap (60) to generate heatmaps. Statistical tests are indicated in the figure legends. Read counts and transcripts per kilobase million (TPM) values per transcript isoform were determined with Salmon version 1.4.0 (61) using Ensembl v104 for GRCh38 as the target transcriptome and the remainder of the genome as decoy. Gene counts were summarized from Salmon transcript isoform estimates using R package tximport version 1.22 (62). Summarized gene counts were normalized and prefiltered to remove genes with consistently low expression across all samples or extreme outliers in 1 or 2 samples, and differential gene expression analysis was performed using DESeq2 v1.34.0 (63) with default values. DESeq2 *P* values were corrected for multiple testing using the Benjamini-Hochberg procedure (64). Differentially expressed genes (DEGs) were defined as an FDR-adjusted *P* value ≤ 0.05 and log_2_FC > 1 or < –1. GSEA was performed with fgsea v1.20 (65) using predefined gene sets from the Molecular Signatures Database (MSigDB v7.5.1). Gene lists were ranked on log_2_FC or with the ashr method (66) using DESeq2. Classical enrichment statistics with 1,000,000 permutations were used to determine significant enrichment within gene sets.

Olink PEA data were analyzed as described previously (35). Ward’s clustering criterion was implemented using the Ward2 algorithm (67, 68). For RNA sequencing and Olink PEA data, the PIBD-SETQuality subcohort was dichotomized into SLPI^low^ (negative IHC) and SLPI^high^ (weak/strong IHC) groups based on the SLPI immunohistochemistry score for the paired biopsy (using the cutoff that resulted in the most equal distribution of patients across 2 groups). Fisher’s exact tests were 2-sided. For details on statistical comparisons, see Supplemental Table 2.

### Study approval.

The PIBD-SETQuality cohort was approved by the Medical Ethical Committee of the Erasmus University Medical Center–Sophia Children’s Hospital Rotterdam (METC number: trial registration number NCT03571373) (34). The IBD longitudinal cohort was approved by the Medical Ethical Committee of the Erasmus University Medical Center–Sophia Children’s Hospital Rotterdam (METC 2007-335). All patients and parents signed informed consent for collection of biomaterials. Murine experiments were approved by the animal experimental committee of the Erasmus Medical Centre, Rotterdam, Netherlands.

### Data availability.

Source data are provided in the Supporting Data Values file. The data used to generate Figure 2, A and B, were derived from publicly available datasets referenced in the article and figure legend. Data, analytic methods, and study materials are available via the repository URL: https://doi.org/10.34894/8YITJ2

## Author contributions

JNS, LDR, and JCE contributed to study concept, design, and funding. JNS supervised the study. SN, YSO, DHHVH, WKS, RCWK, BT, DJLK, and MFP contributed to patient recruitment, clinical data registration, experimental immunological analyses, and clinical data interpretation. BC, MAS, and GVB performed RNA analyses. SN and MD performed histological disease activity scoring. BC and MC performed proteomic analysis. SN, BC, and JNS performed data analyses, had full access to study data, and take responsibility for data integrity. SN, MFP, BC, and JNS drafted the manuscript. All authors contributed to critical revision of the manuscript, provided important intellectual content, and approved the final version of the manuscript. Authorship order among co-first authors was determined based on the extent of their contributions to the work.

## Conflict of interest

The authors have declared that no conflict of interest exists.

## Funding support

- Dutch Digestive Foundation (grant number: Focus Project 15-17).
- PIBD Network for Safety, Efficacy, Treatment and Quality improvement of care project funded by the European Commission Horizon 2020 (grant 668 023).
- European Union’s Horizon 2020 Research and Innovation Programme under the Marie Skłodowska-Curie (grant 95532).
- The Stichting Dalijn.
- Dutch Sophia Foundation (grant 21-45).
- Collaborative T Cell–Driven Immune Mediated Inflammatory Diseases (TIMID) project (grant LSHM18057-SGF) financed by the public-private partnership (PPP) allowance made available by Top Sector Life Sciences & Health to Samenwerkende Gezondheidsfondsen (SGF) to stimulate public-private partnerships and cofinancing by health foundations that are part of the SGF.

## Supplementary Material

Supplemental data

Supporting data values

## Version 1 03/10/2026

In-Press Preview

## Version 2 04/22/2026

Electronic publication
