# Supplementary material for: Epithelial SLPI expression in severe inflammatory bowel disease relates to high IL-17 and neutrophil programming
Source: JCI Insight. 2026 Mar 10;11(8):e197126. doi: 10.1172/jci.insight.197126 (PMC13135391; doi:10.1172/jci.insight.197126)
Supplement: Supplemental data [file jciinsight-11-197126-s153.pdf]

Supplemental Methods

Quantitative PCR (qPCR)

Total RNA was extracted from tissues using the NucleoSpin RNA extraction kit for isolation of RNA (Macherey-Nagel). RNA quantity and purity were assessed using a DeNovix DS-11 spectrophotometer. A maximum of 1000 nanograms of mRNA was used to synthesize cDNA with the SensiFAST cDNA Synthesis Kit (Bioline). Real-time qPCR was performed on a CFX96 Touch Real-Time PCR Detection System (Bio-Rad) using the SensiMix™ SYBR® Hi-ROX Kit (Bioline) and the primers listed in the table below. Relative expression was calculated as $2^{-(\text{Ct value housekeeping gene} - \text{Ct value gene of interest})}$ using the housekeeping genes glyceraldehyde 3-phosphate dehydrogenase (*GAPDH*) for human targets and cyclophilin A for murine targets.

Gene	Forward Primer	Reverse Primer
GAPDH (human)	GTCGGAGTCAACGGATT	AAGCTTCCCGTTCTCAG
SLPI (human)	TCCAGGGAAGAAGAGATGT	TGCCCATGCAACACTT
S100A8 (human)	GTTGACCGAGCTGGAGA	GCACCCTTTTTCCTGATATAC
S100A9 (human)	TCCTCGGCTTTGACAGA	CGCACCAGCTCTTTGA
Cyclophilin A (murine)	AACCCCACCGTGTTCT	CATTATGGCGTGTAAAGTCA
Slpi (murine)	GTGACGGCAAATACAAGTG	GAGCCAAAAGGAGATGTTAGT

Haematoxylin & eosin staining

Sections were deparaffinized in xylene, rehydrated in ethanol, stained with haematoxylin (Vector Laboratories; 7 minutes), stained with Eosin Yellowish (Sigma-Aldrich; 7 minutes, dehydrated in xylene, and mounted in Entallan™ (Sigma-Aldrich).

Measurement of human SLPI protein in supernatant

Human SLPI in TR146 cell supernatants was quantified using an enzyme-linked immunosorbent assay (ELISA). A 96-well high binding surface flat-bottom plate (Corning®; #9018) was coated with 0.5 µg/mL monoclonal antibody against human SLPI (R&D Systems/Bio-Techne; MAB1274; clone 20409) in PBS overnight at room temperature, washed with 0.05% Tween 20 (Sigma-Aldrich) in PBS, blocked with 10% fetal calf serum (Bodinco) in PBS for 1 hour at room temperature, then incubated with samples for 2 hours at room temperature. Standard curves were generated using recombinant human SLPI (R&D Systems/Bio-Techne; 1274-PI-100; 31.25–2000 pg/mL). To detect SLPI, the plate was incubated with 0.1 µg/mL biotinylated polyclonal antibody against human SLPI (R&D Systems/Bio-Techne; BAF1274) for 1 hour at room temperature, followed by 1:5000 horseradish peroxidase-conjugated streptavidin (BD Biosciences; #554066) in the dark at room temperature for ~1 hour. 3, 3′,5 ,5′-Tetramethylbenzidine Liquid Substrate (Sigma-Aldrich; T4444) was used to detect horseradish peroxidase, the reaction was stopped using 1 M H_3PO_4, and signals were detected at 450 nm (570 nm background) using a VersaMax reader (Molecular Devices).

Dextran sodium sulphate (DSS)-induced colitis

A total of 11 C57/Bl6 wildtype mice (both males and females) received 2% dextran sodium sulphate (TdB Consultancy) in drinking water ad libitum for 5 days in two independent experiments; three C57/Bl6 wildtype littermates were untreated, as described in the Methods section. Mice were reared under specific pathogen-free conditions. Faecal samples were collected daily from day 1 until day 10, placed on ice, and stored at -80°C. Mice were sacrificed on day 5, 10, or 36. Distal colonic tissue was fixed in 4% paraformaldehyde for 4 hours at room temperature, stored in 70% ethanol at 4°C for 12 hours to 3 weeks, paraffin embedded, and 4-micrometre-thick sections were mounted on polysine adhesion glass slides (Thermo Fisher Scientific). Adjacent distal colonic tissue was placed in RNAlater® (Sigma-

Aldrich) overnight at 4°C and stored at -80°C. DSS-induced intestinal damage was scored using the scoring system for inflammation-associated histological changes during DSS-induced colitis described by Wirtz et al. (1). Animal experiments were approved by and performed in accordance with the animal experimental committee of the Erasmus Medical Centre.

RNA sequencing of biopsies

One ileal and one colonic biopsy were selected per patient based on the highest modified GHAS in the paired biopsy, as described in the Methods section. If the quality of the RNA extracted from that biopsy was insufficient (see below), the biopsy from the region with the second-highest modified GHAS in the paired biopsy was used.

RNA quality was assessed by capillary gel electrophoresis. Briefly, RNA was inserted into an Agilent 6000 Nano Chip (25-250 ng/µL; when >250 ng/µL samples were diluted) and loaded into the BioAnalyzer (Agilent, Santa Clara, CA, United States) according to the manufacturer's instructions. An appropriate eukaryotic total RNA assay was chosen and the 2100 Expert software (Agilent) was used to calculate the RNA integrity number (RIN). If the RNA concentration was below 25 ng/µL or the RIN could not be calculated, RNA was inserted into an Agilent 6000 Pico Chip (250-5000 pg/µL; when >5000 pg/µL samples were diluted) and the RIN was calculated again. Samples with a RIN < 6 were excluded.

Library preparation was performed on 500 ng total RNA using the KAPA mRNA HyperPrep Kit (Roche) according to the manufacturer's instructions. Briefly, mRNA was captured via hybridization to oligo(dT)-conjugated magnetic beads, washed, eluted, fragmented at high temperature in a magnesium-containing solution, primed with random primers, and reverse transcribed, followed by a second strand synthesis. The dsDNA molecules were tailed with deoxyadenosine at the 3` ends, adapters were ligated to the overhangs, and the DNA was precipitated using magnetic beads and washed with ethanol. After elution of DNA, further amplification via PCR was performed using primers that anneal to the adapters, flanked by

Illumina P5 and P7 sequences. The concentration and size distribution of the libraries were measured using the Qubit ds HS Assay (Thermo Fisher Scientific) and High Sensitivity DNA Kit for BioAnalyzer (Agilent), respectively. Libraries were diluted to 2 nM and paired-end sequenced on a Novaseq 6000 (Illumina) with 2 x 100 read-length at an expected library size of approximately 40 million reads per sample.

Differential expression analysis, pathway analysis and statistical approaches are defined in the main Methods section. The gene set "IL-17 signature" shown in Figure 4f was compiled using the following sources: the 'IL-17 Family Signaling Pathways SuperPath' from PathCards (2); the C2 curated gene sets 'AUJLA_IL22_AND_IL17A_SIGNALING' (M6335) (3), 'BIOCARTA_IL17_PATHWAY' (M19422) (4), REACTOME_INTERLEUKIN_17_SIGNALING' (M27382; R-HSA-448424) (5), 'WP_IL17_SIGNALING_PATHWAY' (M39560) (6); and the C5 collection Gene Ontology gene sets (7) 'GOBP_INTERLEUKIN_17_PRODUCTION (M23117), 'GOBP_POSITIVE_REGULATION_OF_INTERLEUKIN_17_PRODUCTION' (M11373), 'GOBP_RESPONSE_TO_INTERLEUKIN_17' (M23117) and 'GOMF_INTERLEUKIN_17_RECEPTOR_ACTIVITY' (M26514) (7-10).

RNA sequencing of neutrophils

Neutrophils were isolated from 500 µL whole blood using the EasySep Direct Human Neutrophil Isolation Kit (STEMCELL Technologies Germany). A negative selection strategy was applied using an antibody cocktail to bind non-neutrophil cells to magnetic beads. Purified neutrophils were directly stored in Lysis Buffer RA1 (Macherey-Nagel) + 20 mM dithiothreitol at -80°C. cDNA was generated from neutrophil lysates with the NucleoSpin RNA XS extraction kit (Macherey-Nagel) and SensiFAST cDNA synthesis kit. cDNA was quantified with the Qubit dsDNA HS Kit (Thermo Fisher Scientific) and 5 µg was sonicated on a Covaris S220, diluted to 52.5 µL in low-TE buffer (10 mM Tris, 0.1 mM EDTA), and fragmented (peak incident power 250, duty factor 10, cycles/burst 200, duration 80), purified,

and concentrated to 15 µL using a DNA Clean & Concentrator-5 column (Zymo Research). All eluted cDNA fragments were applied as input to the ssDNA Library Prep Kit (Integrated DNA Technologies); DNA:RNA hybrids were denatured and an adapter was ligated to the 3` end of the ssDNA strands. Following polymerase extension, a second adapter was ligated to the 5` end of the original strand. A sequencing library was generated from the adapters via ten cycles of indexing PCR using unique dual index primer pairs for every sample. Library concentrations were measured using the Qubit dsDNA HS kit; the 2100 BioAnalyzer with DNA High Sensitivity Kit (Agilent Technologies) confirmed average library size distribution was ~425 base pairs. Post-library prep ribodepletion was performed using the SEQuoia RiboDepletion Kit (Bio-Rad). The rRNA-derived DNA amplicons in a 20 ng cDNA library were hybridized to sequence-specific biotinylated probes, followed by elongation of the probes via DNA polymerase to strengthen the hybridization. Streptavidin-coated magnetic beads were used to pull down the probes and their target molecules. The remaining library was further PCR-amplified over eight cycles with P5/P7-Illumina-compatible primers. Concentrations and library size distribution were determined before proceeding to the Novaseq 6000 as described in "RNA sequencing of biopsies".

Supplemental Figure 1:

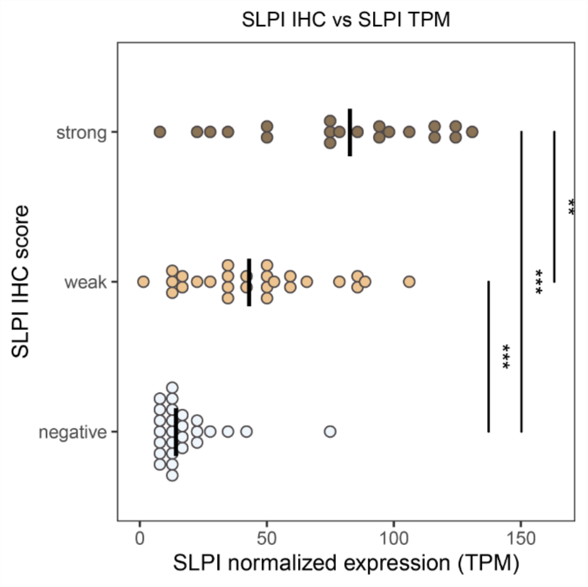

Supplemental Figure 1: Relationship between SLPI protein detection by immunohistochemistry (IHC scores) and SLPI mRNA expression supports the reliability of semiquantitative scoring

Colonic biopsies were classified as negative, weak or strong based on semiquantitative SLPI immunohistochemistry (IHC) scoring. Relative expression (TPM) of SLPI mRNA in paired biopsies was determined to demonstrate a significant relationship between SLPI IHC and mRNA expression. Wilcoxon rank sum test; *: $P < 0.05$, **: $P < 0.01$, ***: $P < 0.001$.

Supplemental Figure 2:

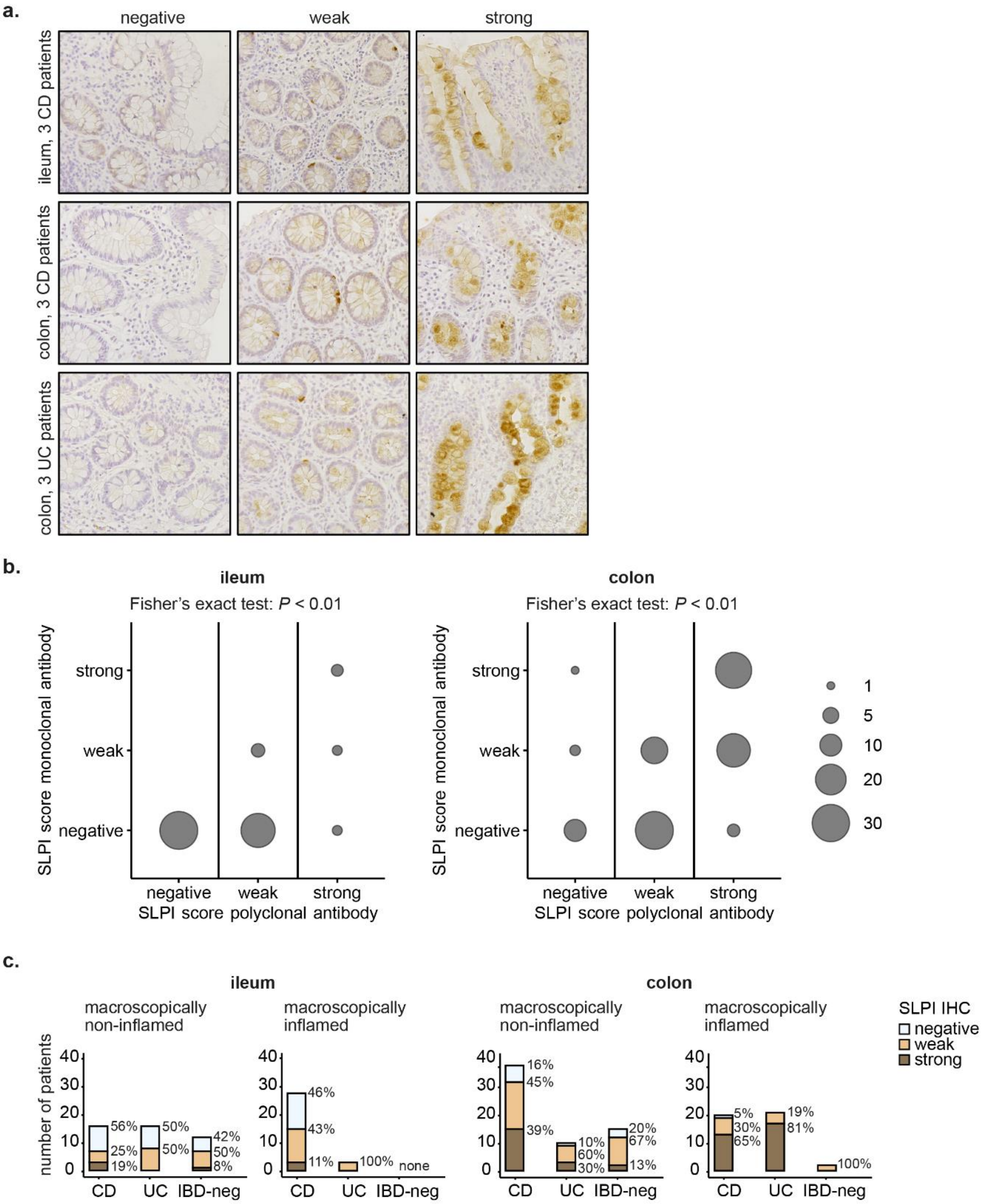

Supplemental Figure 2: Relationship between SLPI immunohistochemistry scores for intestinal biopsies stained with the monoclonal and polyclonal antibodies

(a + b + c) SLPI protein expression was detected by immunohistochemistry (IHC) using the polyclonal antibody in macroscopically non-inflamed and macroscopically inflamed ileal and colonic biopsies from patients with CD (*n* = 41), patients with UC (*n* = 22), and IBD-negative controls (*n* = 15) from the PIBD-SETQuality subcohort. (a) Representative images were acquired at 20x magnification. The intensity of epithelial SLPI staining was scored in a semi-quantitative manner. (b) The relationship between the SLPI scores of biopsies stained with the monoclonal and polyclonal antibodies was tested for all ileal biopsies (*n* = 76) and all colonic biopsies (*n* = 114) using Fisher's exact test. (b) The size of the circles represents the number of biopsies. (c) Distribution of the maximum SLPI score per macroscopically non-inflamed and inflamed ileum and colon biopsy. (c) Percentages of patients with each SLPI IHC score per group.

Supplemental Figure 3:

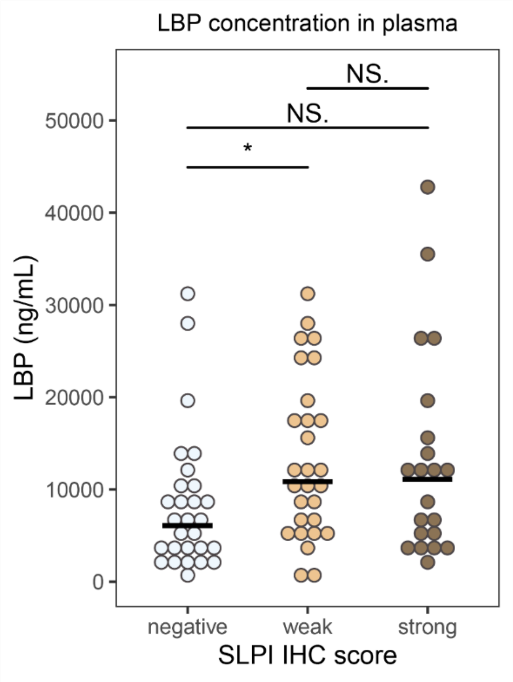

Supplemental Figure 3: Increased plasma lipopolysaccharide-binding protein concentrations in patients with weak or strong colonic SLPI expression

Lipopolysaccharide-binding protein (LBP) concentrations in plasma of IBD patients and controls at diagnosis classified by their colonic SLPI immunohistochemistry (IHC) score (negative, weak, strong). Wilcoxon rank sum test; *: $P < 0.05$, **: $P < 0.01$, ***: $P < 0.001$.

Supplemental Figure 4:

a.

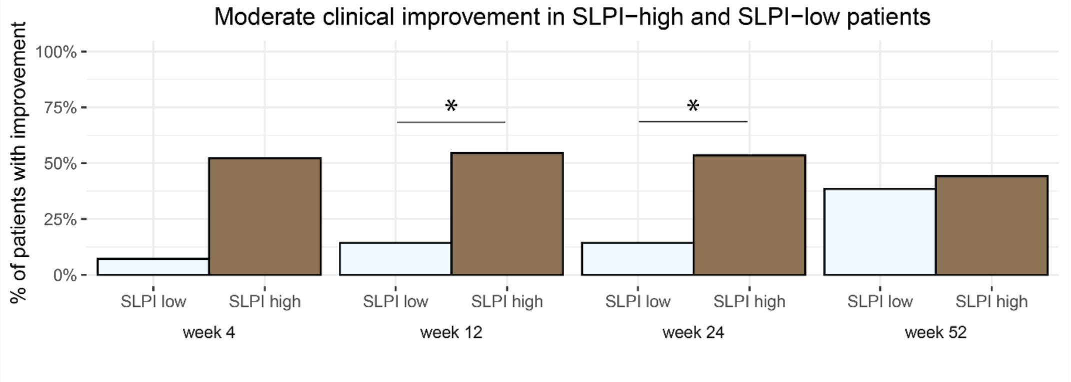

b.

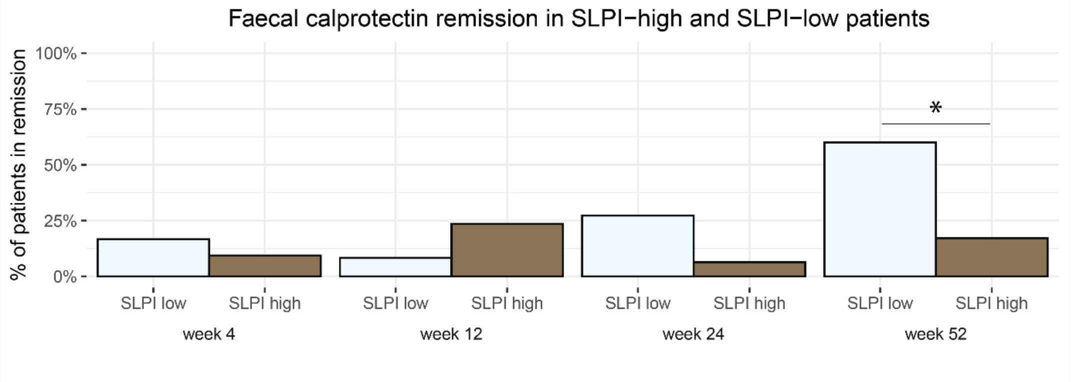

Supplemental Figure 4: Improvement in clinical disease activity and faecal calprotectin remission over one year in patients with low or high colonic epithelial SLPI expression

(a) Percentage of patients having a moderate clinical improvement (>37.5 decrease in wPCDAI (for CD) / ≥ 30 decrease in PUCAI (for UC) compared to scores at diagnosis) at the given timepoints. Fisher's exact tests were performed to assess associations between SLPI expression and clinical improvement status. (b) Percentage of patients achieving faecal calprotectin (FCP) remission (clinical remission with FCP <300 µg/g) at different timepoints. Fisher's exact tests were performed. (a+b) Patients are classified according to low or high colonic epithelial SLPI expression; the timepoint is indicated by weeks after diagnosis. *: $P < 0.05$.

Supplemental Figure 5:

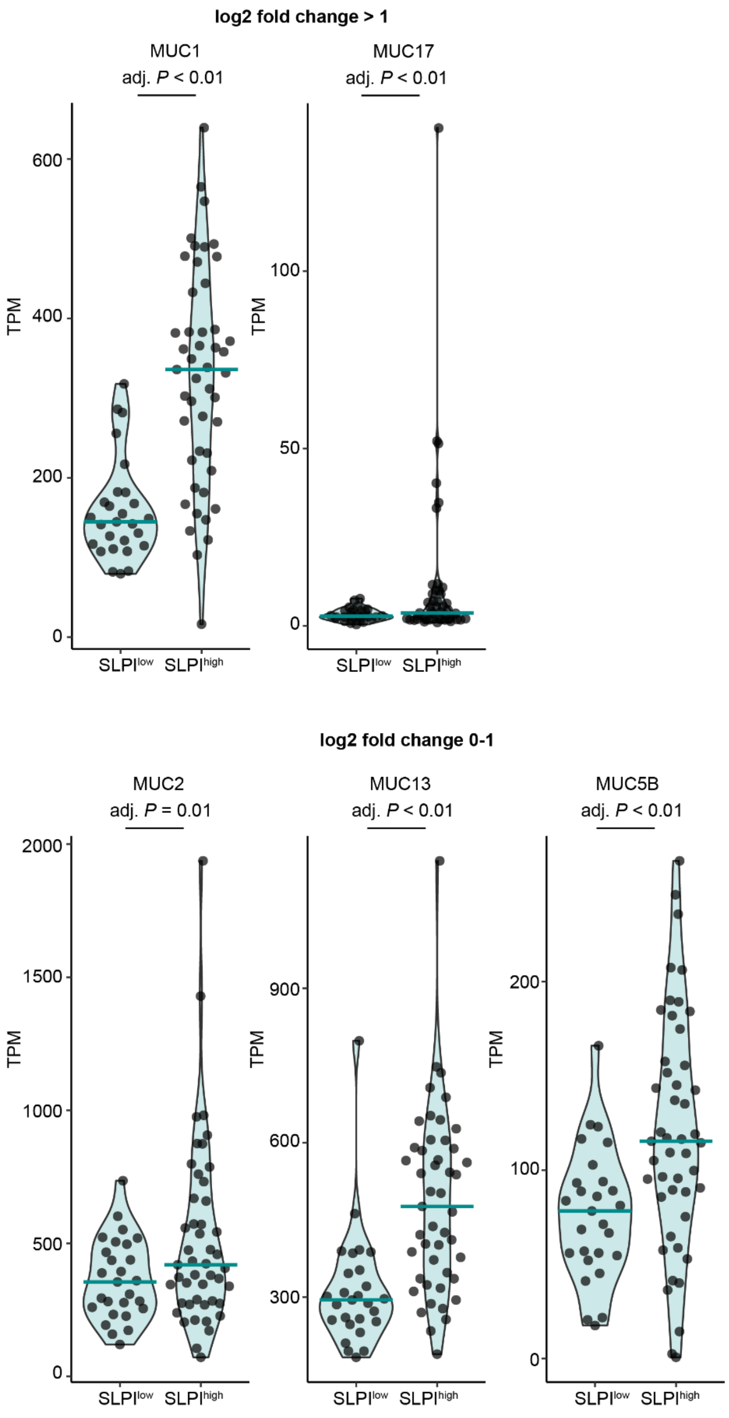

Supplemental Figure 5: Expression of genes encoding mucins is upregulated in the paired colonic biopsy from patients with high SLPI immunohistochemistry scores

RNA sequencing was performed on the paired biopsy from the most affected colonic region per patient (as explained in Figure 4a) from the PIBD-SETQuality subcohort, including patients with CD, patients with UC, and IBD-negative controls. Patients were classified into $SLPI^{low}$ and $SLPI^{high}$ groups based on the SLPI immunohistochemistry (IHC) score. Gene expression was compared between the $SLPI^{high}$ group and $SLPI^{low}$ group using DESeq2 followed by Benjamini-Hochberg correction of *P*-values. Genes were considered differentially expressed if the log2 fold change was > 1 or < -1 and the adjusted *P*-value was < 0.05. Violin plots of genes encoding mucins for colonic biopsies from the $SLPI^{low}$ and $SLPI^{high}$ groups.

Supplemental Figure 6:

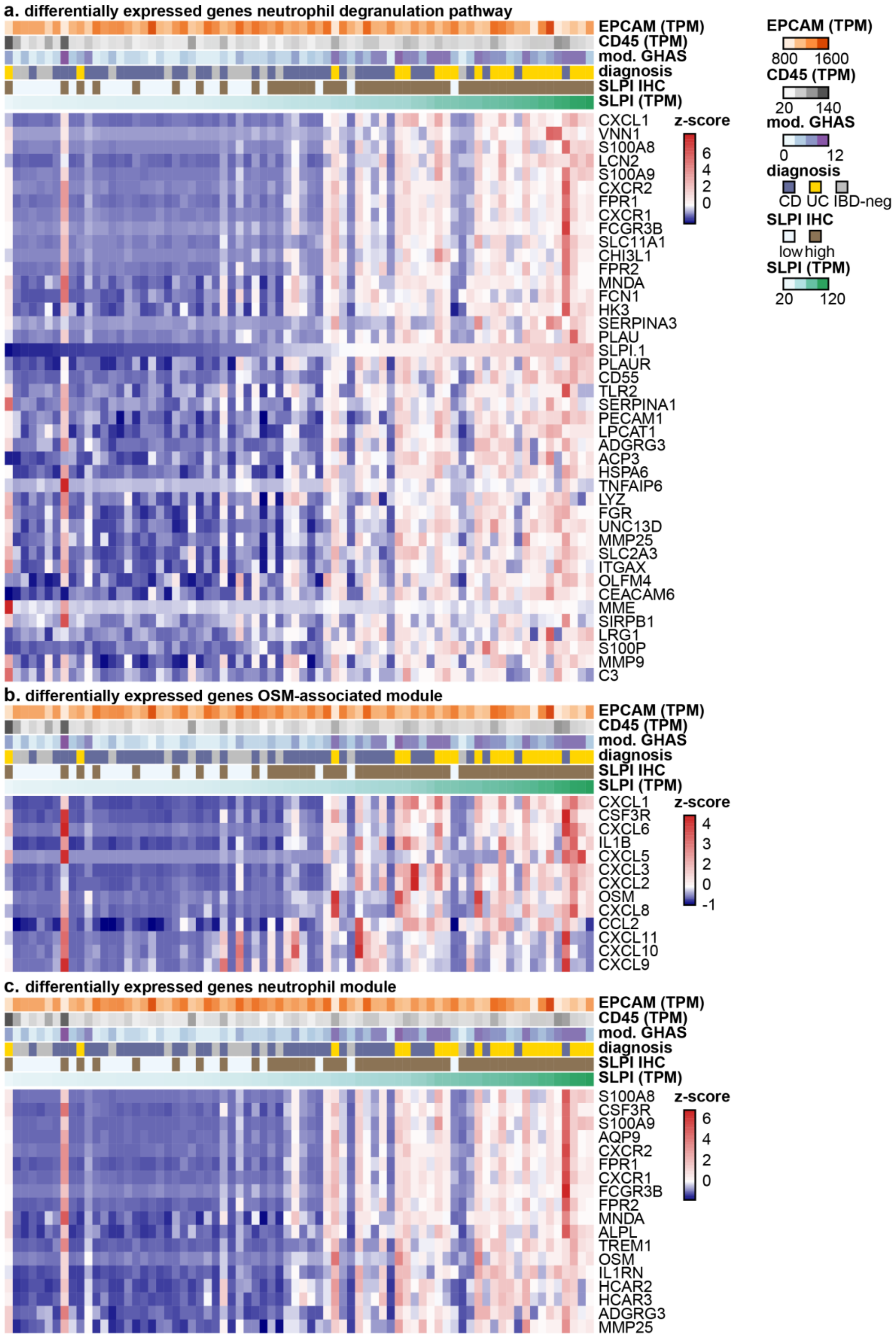

Supplemental Figure 6: Neutrophil related genes are upregulated in the paired colonic biopsy from patients with high SLPI immunohistochemistry scores

(a + b + c) RNA sequencing was performed on the paired biopsy from the most affected colonic region per patient (as explained in Figure 4a) in the PIBD-SETQuality subcohort, including patients with CD, patients with UC, and IBD-negative (IBD-neg) controls. Patients were classified into $SLPI^{low}$ and $SLPI^{high}$ groups based on the SLPI immunohistochemistry (IHC) score. Gene expression was compared between the $SLPI^{high}$ and $SLPI^{low}$ groups using DESeq2 followed by Benjamini-Hochberg correction of *P*-values. Genes were considered differentially expressed if the log2 fold change was > 1 or < -1 and the adjusted *P*-value < 0.05. (a + b + c) The heatmaps show z-scored TPM values for differentially expressed genes in colonic biopsies from the $SLPI^{high}$ group versus the $SLPI^{low}$ group. (a + b + c) The genes are ranked according to significance (lowest adjusted *P*-value on top) and the samples are ranked according to SLPI TPM values (highest TPM value on right). The gene set 'neutrophil degranulation pathway' (a) was derived from the C2 curated gene set 'REACTOME_NEUTROPHIL_DEGRANULATION' (M27620) (5). The gene set 'OSM-associated module' (b) was derived from West et al. (11). The gene set 'neutrophil module' (c) was derived from Friedrich et al. (12).

Supplemental Figure 7

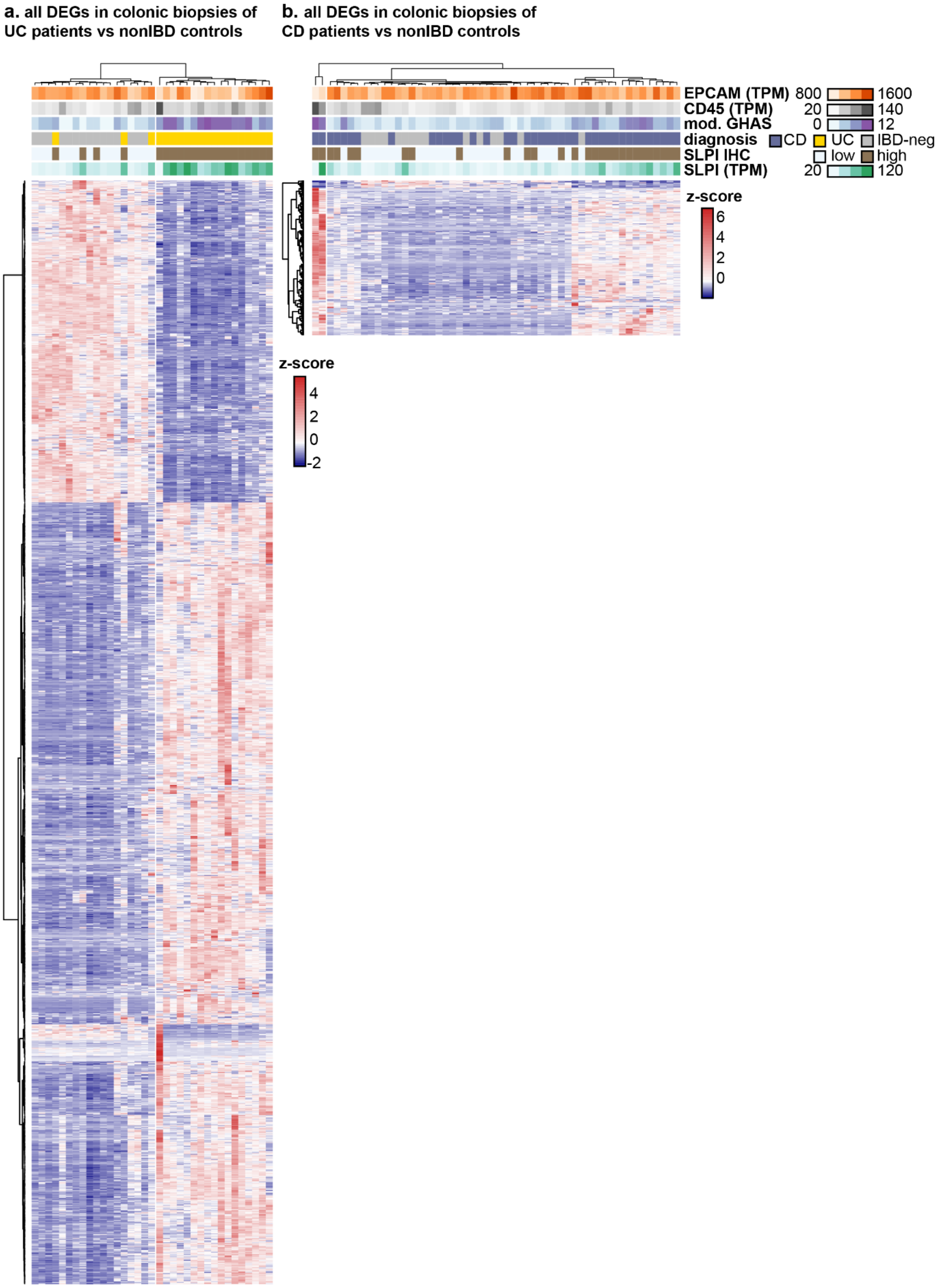

Supplemental Figure 7: Unbiased hierarchical clustering of patients on the basis of their colonic transcriptome groups patients in clusters with high or low SLPI protein expression on immunohistochemistry.

Two adjacent (paired) biopsies were collected from multiple colonic regions during endoscopy for therapy-naive patients with CD, patients with UC, and IBD-negative (IBD-neg) controls from the PIBD-SETQuality subcohort. One of the paired biopsies was used for histological analysis, including the modified GHAS, which was used to define the most affected colonic region per patient. Gene expression of the most affected biopsies was compared between UC (n=21) versus IBD-neg (n=15) (a) or CD (n=51) versus IBD-neg (n=15) (b) and differentially expressed genes (DEGs) were defined as a FDR-adjusted *P*-value ≤ 0.05 and log2FC > 1 or < -1. (a) UC versus IBD-neg n=1510 DEGs; (b) CD versus IBD-neg n=215 DEGs. (a + b) Next, hierarchical clustering using Ward's criterion on the basis of these DEGs was performed to assess relationships with SLPI immunohistochemistry (IHC) scores in the paired biopsy ($SLPI^{low}$ = negative IHC and $SLPI^{high}$ =weak/strong IHC). Above the heatmap, TPM values for EPCAM (as a measure of an epithelial signal), CD45 (a hematopoietic cell signal), the SLPI IHC dichotomous score (used to perform differential expression analysis), the modified GHAS score from the paired biopsy, and the diagnosis are shown.

Supplemental Figure 8:

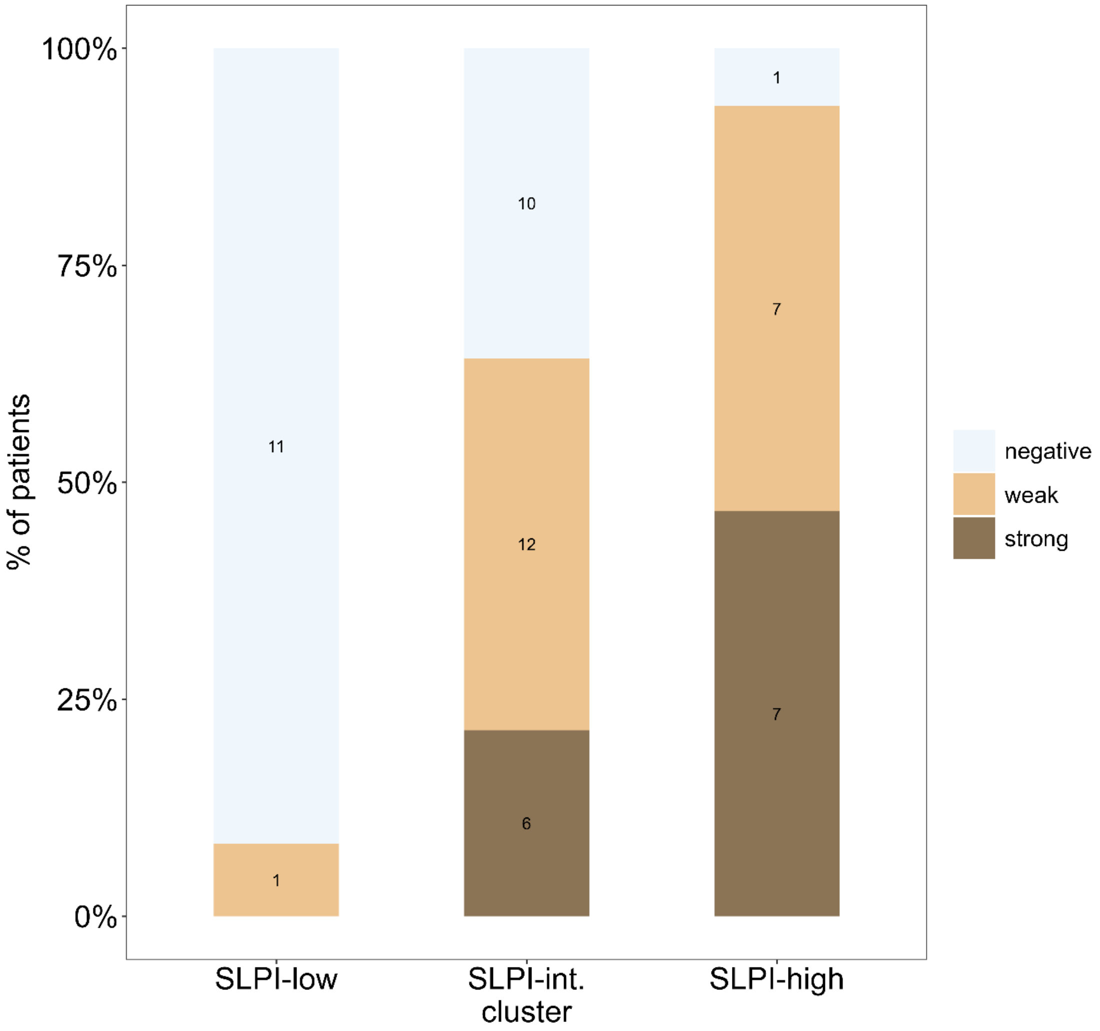

Supplemental Figure 8: SLPI protein expression as detected by immunohistochemistry in colonic biopsies of patients clustered according to their neutrophil transcriptome in figure 6c.

IBD patient clusters were identified by hierarchical clustering on the basis of differentially expressed genes in circulating neutrophils from SLPI^{high} versus SLPI^{low} patients, as described in Figure 6c. SLPI immunohistochemistry (IHC) scores for colonic biopsies ranging from negative, weak to strong are shown for the three neutrophil transcriptome patient clusters: the ‘SLPI-high’ cluster, ‘SLPI-low’ cluster, and ‘SLPI-intermediate’ cluster. The numbers in the bars represent the number of patients in each group. Fisher’s exact test: *P*-value < 0.01.

Supplemental Table 1: Sera and antibodies used for immunohistochemistry

Protein	Serum	Primary Antibody	Secondary Antibody
Human SLPI*	Normal horse serum (Biowest)	Anti-human SLPI monoclonal mouse IgG1 antibody, 4 µg/mL (HycultBiotech; HM2037, clone 31)	Horse-anti-mouse antibody, 1:500 (Vector Laboratories)
Human SLPI	Normal rabbit serum (Jackson ImmunoResearch)	Anti-human SLPI polyclonal goat IgG antibody, 1 µg/mL (R&D Systems/Bio-Techne; BAF1274)	Rabbit anti-goat antibody, 1:500 (Vector Laboratories)
Human S100A8/A9	Normal horse serum (Biowest)	Anti-human S100A8/S100A9 heterodimer monoclonal mouse IgG1 antibody, 1.2 µg/mL (R&D Systems/Bio-Techne; MAB45702)	Horse anti-mouse antibody, 1:500 (Vector Laboratories)
Human IL-17	Normal rabbit serum (Jackson ImmunoResearch)	Anti-human IL-17/IL-17A polyclonal goat IgG antibody, 2 µg/mL (R&D Systems/Bio-Techne; AF-317)	Rabbit anti-goat antibody, 1:500 (Vector Laboratories)
Murine SLPI	Normal rabbit serum (Jackson ImmunoResearch)	Anti-mouse SLPI polyclonal goat IgG antibody, 2 µg/mL (R&D Systems/Bio-Techne; AF1735)	Rabbit anti-goat antibody, 1:500 (Vector Laboratories)

*The monoclonal antibody was used to detect SLPI, unless otherwise indicated.

Supplemental Table 2: Additional statistical comparisons for main figures in the results section

Figure	Comparison	*P*-value	Statistical test
2a	Microarray SLPI expression<br>Ileum vs colon	$P < 0.01$	Wilcoxon rank sum test
2b	Microarray SLPI expression<br>Ileum vs colon	$P < 0.01$	Wilcoxon rank sum test
2c	Relative SLPI expression<br>Ileum vs colon	$P < 0.01$	Wilcoxon rank sum test
2f	SLPI and diagnosis<br>negative vs weak	$P = 0.27$	Fisher's exact test
2f	SLPI and diagnosis<br>weak vs strong	$P = 0.23$	Fisher's exact test
2f	SLPI and diagnosis<br>negative vs strong	$P = 0.02$	Fisher's exact test
2f	SLPI and clinical disease activity<br>negative vs weak	$P = 0.53$	Fisher's exact test
2f	SLPI and clinical disease activity<br>weak vs strong	$P = 0.03$	Fisher's exact test
2f	SLPI and clinical disease activity<br>negative vs strong	$P = 0.01$	Fisher's exact test
2g	SLPI and SES-CD<br>negative vs weak	$P < 0.01$	Kruskal-Wallis rank sum test
2g	SLPI and SES-CD<br>weak vs strong	$P = 0.79$	Kruskal-Wallis rank sum test
2g	SLPI and SES-CD<br>negative vs strong	$P < 0.01$	Kruskal-Wallis rank sum test
2g	SLPI and UCEIS<br>negative vs weak	$P = 0.09$	Kruskal-Wallis rank sum test
2g	SLPI and UCEIS<br>weak vs strong	$P = 0.39$	Kruskal-Wallis rank sum test
2g	SLPI and UCEIS<br>negative vs strong	$P = 0.09$	Kruskal-Wallis rank sum test
2g	SLPI and total UCEIS<br>negative vs weak	$P = 0.08$	Kruskal-Wallis rank sum test
2g	SLPI and total UCEIS<br>weak vs strong	$P = 0.54$	Kruskal-Wallis rank sum test
2g	SLPI and total UCEIS<br>negative vs strong	$P = 0.03$	Kruskal-Wallis rank sum test

Figure	Comparison	P value	Statistical test
3a	SLPI and modified GHAS negative vs weak	*P* < 0.01	Kruskal-Wallis rank sum test
3a	SLPI and modified GHAS weak vs strong	*P* = 0.07	Kruskal-Wallis rank sum test
3a	SLPI and modified GHAS negative vs strong	*P* < 0.01	Kruskal-Wallis rank sum test
3a	SLPI and activity GHAS negative vs weak	*P* < 0.01	Kruskal-Wallis rank sum test
3a	SLPI and activity GHAS weak vs strong	*P* < 0.01	Kruskal-Wallis rank sum test
3a	SLPI and activity GHAS negative vs strong	*P* < 0.01	Kruskal-Wallis rank sum test
3c	SLPI and calprotectin negative vs weak	*P* < 0.01	Fisher's exact test
3c	SLPI and calprotectin weak vs strong	*P* = 0.17	Fisher's exact test
3c	SLPI and calprotectin negative vs strong	*P* < 0.01	Fisher's exact test
5b	SLPI and mononuclear cells negative vs weak	*P* < 0.01	Fisher's exact test
5b	SLPI and mononuclear cells weak vs strong	*P* = 0.36	Fisher's exact test
5b	SLPI and mononuclear cells negative vs strong	*P* < 0.01	Fisher's exact test
5c	SLPI and IL-17 negative vs weak	*P* < 0.01	Kruskal-Wallis rank sum test
5c	SLPI and IL-17 weak vs strong	*P* = 0.90	Kruskal-Wallis rank sum test
5c	SLPI and IL-17	*P* < 0.01	Kruskal-Wallis rank sum test

Supplemental Table 3:

Clinical variable	SLPI IHC negative (*n* = 15)	SLPI IHC weak (*n* = 27)	SLPI IHC strong (*n* = 20)	Total (*n* = 62)	*P*-value
Age at diagnosis in years, mean (SD)	13.4 (2.5)	13.9 (2.2)	13.9 (2.5)	13.8 (2.3)	0.72[1]
Sex					0.85[2]
• Female	8 (53%)	12 (44%)	10 (50%)	30 (48%)	
• Male	7 (47%)	15 (56%)	10 (50%)	32 (52%)	
Diagnosis					**0.04[2]**
• CD	13 (87%)	18 (67%)	9 (45%)	40 (65%)	
• UC	2 (13%)	9 (33%)	11 (55%)	22 (35%)	
Disease activity					**0.01[2]**
• None or mild	9 (60%)	13 (48%)	3 (15%)	25 (40%)	
• Moderate to severe	6 (40%)	14 (52%)	17 (85%)	37 (60%)	
SES-CD in patients with CD, mean (SD)	6.4 (2.6)	15.4 (7.3)	16.3 (6.9)	12.1 (7.3)	**<0.01[1]**
UCEIS in patients with UC, mean (SD)	2.5 (0.7)	4.1 (1.1)	4.9 (1.9)	4.4 (1.6)	0.14[1]
Total UCEIS in patients with UC, mean (SD)	2.5 (0.7)	14.4 (6.4)	18.0 (8.8)	15.0 (8.4)	0.09[1]
Disease location in CD patients					**<0.01[3]**
• Ileal	12 (92%)	6 (33%	2 (22%)	20 (50%)	
• Colonic	0 (0%)	2 (11%)	1 (11%)	3 (8%)	
• Ileocolonic	1 (8%)	10 (56%)	6 (67%)	17 (43)	
Disease behaviour in CD patients					0.88[3]
• Non-stricturing or penetrating	10 (80%)	15 (83%)	8 (89%)	33 (83%)	

• Stricturing	3 (39%)	2 (11%)	1 (11%)	6 (15%)	
• Penetrating	0 (0%)	1 (6%)	0 (0%)	1 (3%)	
Disease extent in patients with UC					0.22[2]
• Ulcerative proctitis or left-sided UC	2 (100%)	3 (33%)	3 (27%)	8 (36%)	
• Extensive UC or pancolitis	0 (0%)	6 (67%)	8 (73%)	14 (64%)	
Presence of granuloma in any of the biopsies from CD patients					0.49[3]
• Yes	4 (31%)	6 (33%)	5 (56%)	15 (38%)	
• No	9 (69%)	12 (67%)	4 (44%)	25 (62%)	
Perianal disease in CD patients					0.10[3]
• Yes	2 (15%)	2 (11%)	1 (11%)	5 (13%)	
• No	11 (85%)	16 (89%)	8 (89%)	35 (88%)	
Faecal calprotectin in µg/g, mean (SD)	1683 (2027)	2122 (1954)	1428 (1335)	1794 (1807)	0.52[1]

[1] Kruskal-Wallis rank sum test. [2] Pearson's Chi-squared test.[3] Fisher's exact test (two-sided).

Supplemental Table 3: Clinical characteristics of the patients with IBD in the PIBD-SETQuality subcohort

Clinical characteristics at diagnostic endoscopy for the patients with CD or UC included in the PIBD-SETQuality subcohort. Patients are grouped according to SLPI immunohistochemistry score for their colonic biopsy with the highest modified Global Histological disease Activity Score (GHAS). Disease location, disease behaviour, and disease extent were defined according to the Paris classification (13). SD = standard deviation. SES-CD = Simple Endoscopic Score for Crohn's Disease. UCEIS = Ulcerative Colitis Endoscopic Index of Severity. The 'total UCEIS' is the sum of the UCEIS for each colonic segment.

Supplemental Table 4

**Neutrophil transcriptome patient clusters (vertical clustering)**	**Neutrophil gene cluster (horizontal clustering)**	**Enriched pathways (according to Pathway analysis; see methods)**	**Individual genes present in the neutrophil gene cluster**
SLPI-high	High expression of genes in “violet” cluster (middle).	Neutrophil granules; defense response to pathogens; neutrophil activation and migration.	S100A8/9/12, MMP9, ARG1, HGF, CD177, IL18R1
SLPI-low	High expression of genes in “green” cluster (top).	No significant pathways. Genes reflect a plethora of biological processes, such as cell adhesion and migration, signal transduction kinases and vesicle trafficking/transport.	SDC2, SPP1, MAP3K15, CACNB4, MOK, BAIAP3
SLPI-intermediate	High expression of genes in “salmon” cluster (bottom).	Inflammasome; IFN-related pathways; neutrophil granules.	IFITIM3, IFI44L, GBP1, LCN2, MMP8, AIM2

Supplemental Table 4: Key transcriptional features driving the definition of the neutrophil transcriptome patient clusters in Figure 6c.

To decipher which biological processes are different in the neutrophil transcriptome per patient cluster (column 1) we performed hierarchical clustering on the neutrophil genes in the heatmap in Figure 6c (horizontal clusters). Neutrophil genes grouped into three clusters denoted as green; violet and salmon (column 2). Gene Ontology enrichment analysis was performed independently for each gene cluster, and predominant pathway categories are shown (column 3). Pathways were considered significant when adjusted *P*-value < 0.05. Individual genes of interest are shown in column 4.

References

1. Wirtz S, Popp V, Kindermann M, et al. Chemically induced mouse models of acute and chronic intestinal inflammation. *Nat Protoc*. 2017;12(7):1295–1309.

2. Belinky F, Nativ N, Stelzer G, et al. PathCards: multi-source consolidation of human biological pathways. *Database (Oxford)*. 2015;2015:10.1093/database/bav006. Print 2015.

3. Aujla SJ, Chan YR, Zheng M, et al. IL-22 mediates mucosal host defense against Gram-negative bacterial pneumonia. *Nat Med*. 2008;14(3):275–281.

4. Rouillard AD, Gundersen GW, Fernandez NF, et al. The harmonizome: a collection of processed datasets gathered to serve and mine knowledge about genes and proteins. *Database (Oxford)*. 2016;2016:10.1093/database/baw100. Print 2016.

5. Gillespie M, Jassal B, Stephan R, et al. The reactome pathway knowledgebase 2022. *Nucleic Acids Res*. 2022;50(D1):D687–D692.

6. Martens M, Ammar A, Riutta A, et al. WikiPathways: connecting communities. *Nucleic Acids Res*. 2021;49(D1):D613–D621.

7. Liberzon A, Birger C, Thorvaldsdóttir H, Ghandi M, Mesirov JP, Tamayo P. The Molecular Signatures Database (MSigDB) hallmark gene set collection. *Cell Syst*. 2015;1(6):417–425.

8. Subramanian A, Tamayo P, Mootha VK, et al. Gene set enrichment analysis: a knowledge-based approach for interpreting genome-wide expression profiles. *Proc Natl Acad Sci U S A*. 2005;102(43):15545–15550.

9. Ashburner M, Ball CA, Blake JA, et al. Gene ontology: tool for the unification of biology. The Gene Ontology Consortium. *Nat Genet*. 2000;25(1):25–29.

10. Gene Ontology Consortium. The Gene Ontology resource: enriching a GOld mine. *Nucleic Acids Res*. 2021;49(D1):D325–D334.

11. West NR, Hegazy AN, Owens BMJ, et al. Oncostatin M drives intestinal inflammation and predicts response to tumor necrosis factor-neutralizing therapy in patients with inflammatory bowel disease. *Nat Med*. 2017;23(5):579–589.

12. Friedrich M, Pohin M, Jackson MA, et al. IL-1-driven stromal-neutrophil interactions define a subset of patients with inflammatory bowel disease that does not respond to therapies. *Nat Med*. 2021;27(11):1970–1981.

13. Levine A, Griffiths A, Markowitz J, et al. Pediatric modification of the Montreal classification for inflammatory bowel disease: the Paris classification. *Inflamm Bowel Dis*. 2011;17(6):1314–1321.
